# H&E to IHC virtual staining methods in breast cancer: an overview and benchmarking

**DOI:** 10.1038/s41746-025-01741-9

**Published:** 2025-07-02

**Authors:** Pascal Klöckner, José Teixeira, Diana Montezuma, João Fraga, Hugo M. Horlings, Jaime S. Cardoso, Sara P. Oliveira

**Affiliations:** 1 Computational Pathology Group, The Netherlands Cancer Institute, Amsterdam, The Netherlands; 2 Faculty of Engineering, University of Porto, Porto, Portugal; 3 Center for Telecommunications and Multimedia, INESC TEC, Porto, Portugal; 4 IMP Diagnostics, Porto, Portugal; 5 Cancer Biology and Epigenetics Group, Research Center of IPO Porto (CI-IPOP), Portuguese Oncology Institute of Porto (IPO Porto), Porto Comprehensive Cancer Center Raquel Seruca (Porto.CCC) & CI-IPOP@RISE (Health Research Network), Porto, Portugal

**Keywords:** Breast cancer, Diagnostic markers, Computer science, Biomedical engineering

## Abstract

Immunohistochemistry (IHC) is crucial for the clinical categorisation of breast cancer cases. Deep generative models may offer a cost-effective alternative by virtually generating IHC images from hematoxylin and eosin samples. This review explores the state-of-the-art in virtual staining for breast cancer biomarkers (HER2, PgR, ER and Ki-67) and benchmarks several models on public datasets. It serves as a resource for researchers and clinicians interested in applying or developing virtual staining techniques.

## Introduction

Cancer is a heterogeneous disease characterised by significant variability in its biological and clinical manifestations. Pathology, the study of disease, plays a central role in cancer diagnosis through the detailed assessment of tissue morphology as well as the evaluation of molecular and immunohistochemical markers^1^. Pathologists provide comprehensive analyses of biopsy and surgical specimens to determine the precise subtype, grade, and severity of tumours, playing a critical role in cancer patients’ care.

In the traditional pathology workflow, thin tissue sections are cut from tumour specimens and mounted on glass slides, requiring a staining step to highlight the structures of interest^1^. The standard staining technique uses a combination of haematoxylin and eosin (H&E) to highlight the nuclei and cytoplasm of cells. A more advanced technique, immunohistochemistry (IHC), highlights the presence of specific proteins in the tissue, using antibodies and enzymatic reactions. A primary antibody binds to the target antigen, followed by a secondary antibody conjugated to an enzyme that reacts with a chromogenic substrate (such as 3,3’-Diaminobenzidine, DAB) to form an insoluble precipitate (brown, in the case of DAB). Its high contrast with the counter-stain, usually haematoxylin, highlights the location of the target antigen^2,3^. Usually, IHC follows the H&E staining and is performed on consecutive tissue sections.

With the advent of digital pathology, glass slides, previously assessed under a microscope, are now scanned into large, high-resolution files, known as whole slide images (WSI), and analysed on a computer screen. This transition to the digital era has created the opportunity to develop computational approaches for automatic image analysis, mainly based on artificial intelligence (AI), for tasks like tumour detection, grading, segmentation, or even survival prediction and novel diagnostic biomarkers discovery^4,5^.

Computational pathology has been transforming traditional pathology practice by integrating AI and WSI analysis techniques^5^. Specifically, regarding the assessment of different tissue dyes, virtual staining is a promising approach to simulate histological staining on screen^6^. It leverages advanced image processing and AI models to digitally replicate conventional histological stains, such as IHC^7^. By virtually mimicking the staining process, this technique enables the generation of multiple digital slides from a single H&E sample (or even an unstained sample), promoting the efficient use of resources (eco-friendly), preservation of tissue specimens, and reduced lab workload and costs. In addition, by giving an indication of the IHC result, virtual staining reduces the turnaround time for diagnoses, potentially allowing for a faster and more accurate assessment of patient cases, just from the standard H&E sample. Moreover, virtual staining allows for the customisation of staining patterns, tailoring the visualisation of several biomarkers in the same tissue sample and uncovering hidden details that might be otherwise challenging to visualise with conventional staining^8^. Virtual staining represents a promising approach that has the potential to transform how pathologists analyse tissue samples. It is expected to play an increasingly significant role in research, diagnostics, and personalised medicine, ultimately enhancing patient care, particularly in resource-limited settings.

From the AI point of view, virtual staining can be seen as an image-to-image translation problem, where the goal is to map from one image domain (source, e.g., H&E) to another image domain (target, e.g., IHC). Therefore, a good fit for such a task are deep generative models (DGM)^9–11^, a class of algorithms that generate new data from training examples. Deep generative models can learn the underlying structure of data and can generate new samples that closely resemble the original data distribution. Additionally, they also allow training from both paired and unpaired data: while training with paired data requires corresponding samples from both domains, unpaired training only needs an individual dataset from each domain. Although pairwise approaches typically exhibit better performance, unpaired techniques might be more suitable for medical images since pairs are not commonly available^11^.

In recent years, there has been an uptrend in research on virtual staining, particularly for breast cancer (BC) IHC biomarkers, due to the well-established role of biomarker status in treatment decisions and also the high societal impact of breast cancer. With the field evolving rapidly, with diverse new methodologies, this review seeks to offer a comprehensive overview of the various stain-to-stain approaches used in virtual staining for the BC immuno clinical biomarker panel: human epidermal growth factor receptor 2 (HER2), oestrogen and progesterone receptors (ER and PgR, respectively) and Ki67. While we outline the current state-of-the-art techniques, a key contribution of this work is a benchmarking study with a unified performance comparison of different virtual staining models using two widely used datasets for BC biomarkers. In addition, we discuss essential considerations for developing virtual staining frameworks, such as target biomarkers, common DGM frameworks, evaluation metrics, and publicly available datasets. This review specifically emphasises H&E to IHC translation models due to their significant potential for clinical applications, given the widespread use of H&E staining and the well-established role of IHC in clinical decision-making. By integrating these elements, we aim to provide a valuable resource for researchers and clinicians advancing virtual staining technology for breast cancer diagnostics.

## Breast cancer and clinical IHC biomarkers

Breast cancer poses a growing global challenge, intersecting health, gender, socioeconomic, and equity issues^12^. In 2022, BC was diagnosed in 2.3 million women, and there were 670,000 breast cancer-related deaths worldwide^13^. It is the leading cause of cancer death among women, and the burden of avoidable deaths disproportionately affects low- and middle-income countries. The World Health Organisation (WHO) has established the Global Breast Cancer Initiative to address the inequities in BC outcomes, with the overall aim of reducing BC by 2.5% per year (which is estimated to save 2.5 million lives over 20 years)^14^. The Initiative has three fundamental pillars: early detection, timely diagnosis, and comprehensive BC management^14^. The second pillar derives from the concept that early clinical detection of BC improves outcomes only when followed by timely pathological diagnosis and the start of high-quality treatment^14^. Computational strategies, namely virtual staining, which enable rapid and precise tissue analysis without traditional IHC processes, hold significant potential for improving the prompt diagnosis of BC, thereby supporting the goals of WHO’s Breast Initiative.

Diagnosing breast cancer dates back 3500 years, when the disease could be detected through its signs and symptoms, such as palpable lumps^15^. Today, BC is seen as a heterogeneous disease characterised by diverse presentation, morphology, molecular features, clinical behaviour and response to therapy^16,17^. Grading and staging are fundamental for case evaluation, with grading assessing tumour differentiation and proliferative activity while staging determines the extent of disease spread, both being critical for prognosis and treatment planning. Additionally, four main molecular subtypes of invasive BC are recognised: luminal A, luminal B, human epidermal growth factor receptor 2 (HER2)-enriched and basal-like^16,17^. These are broad categories, and subsequent investigations have demonstrated further subclasses^17,18^.

**Fig. 1:**
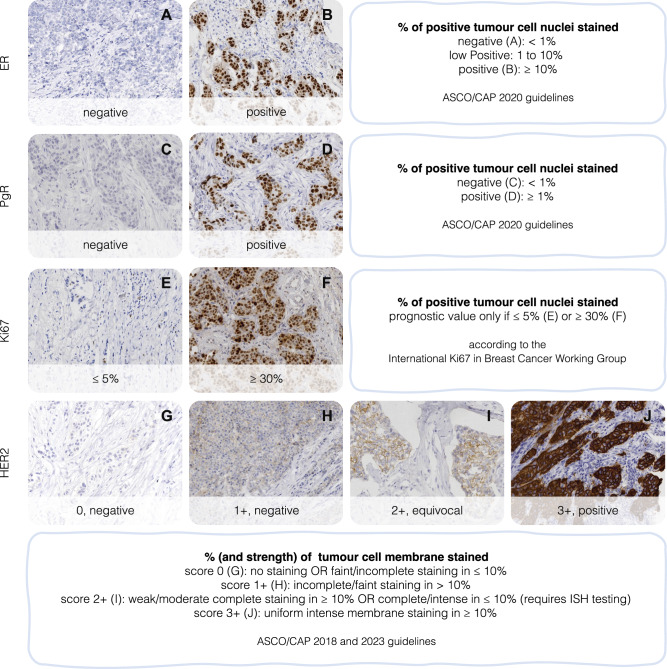
Interpretation of commonly used immunohistochemistry (IHC) markers in breast cancer. **A**, **B** Estrogen receptor (ER); **C**, **D** Progesterone receptor (PgR); **E**, **F** Ki67; **G**–**J** Human epidermal growth factor receptor 2 (HER2). For HER2, an IHC 2+ score requires confirmation by in situ hybridisation (ISH), and an IHC 1+ or 2+ may be reported as “HER2 Low” and are eligible for clinically appropriate HER2-targeted therapy, according to ASCO/CAP 2023 Guideline update. Images adapted from the MIST test set^44^.

Nowadays, the determination of the molecular subtype is not seen as critical in clinical practice, as treatment management is based on traditional clinico-pathologic prognostic and predictive factors (only supplemented by gene expression signature profiling in some cases)^19–22^. Specifically, the immuno-expression profile can assist in the clinical categorisation of invasive carcinoma subtypes. Thus, a combination of immuno-stains is used routinely on breast cancer cases, namely for oestrogen and progesterone receptors (ER and PR, respectively) and HER2, with some sites also using Ki67. The recognised clinical groups are based on the multiple combinations of immuno-expression (for example, the “triple negative” clinical group, i.e., ER/PgR/HER2 negative, or the “HER2+/RH-” group with corresponding immuno-expression). Evaluating the expression of these stains, an essential component of the diagnostic process of all BC patients, is determined using standardised protocols (Fig. 1), following established guidelines^23–26^. Cancer multi-gene prognostic tests can provide additional risk stratification in this setting (such as OncotypeDX, MammaPrint or Prosigna)^27,28^.

Multiple studies have demonstrated hormone receptors (HR) and HER2 to have prognostic and predictive value^29^. When evaluating adjuvant therapy for BC, a key consideration is the tumour’s likelihood of responding to endocrine treatment or HER2-targeted therapy. Tumour ER positivity, found in 75–80% of patients, predicts response to hormone therapy, whereas ER-negative cases will not benefit from it^30,31^. PgR has also been shown to have additional prognostic and predictive relevance in BC^32^. It has a more variable expression profile than ER, having a role in stratifying ER-positive cases into prognostic classes^29^. A cut-off of 1% is generally used to define HR positivity and assess eligibility for hormone therapy, even though the intensity and percentage vary (weak to strong intensity and from 1% to 100% of positive cells within a tumour). Breast cancer cases showing HR strong diffuse nuclear expression usually present better response to endocrine therapy and more favourable outcomes. The HER2 status can be assessed using IHC and/or in situ hybridisation (ISH) techniques, and it determines the response to anti-HER2 therapies^33^. Historically, HER2 overexpression was associated with a worse prognosis. However, with the advent of targeted therapies, survival rates have significantly increased in HER2-positive breast cancer^33^. The International Ki67 in Breast Cancer Working Group has recently concluded that Ki67, a proliferation biomarker, is also a prognostic marker for BC, but its clinical value is limited to ER+/HER2− patients, serving to stratify the cases from this group, which will benefit from adjuvant chemotherapy^34^. Nevertheless, the validity of Ki67 remains questionable due to the poor consensus on scoring methods and cut-off thresholds and concerns regarding the reliability of various antibodies^25^.

Finally, it is worth mentioning that the prognostic assessment of BC is not limited to IHC/molecular panels, as histologic subtype, tumour stage, presence/absence of lymphovascular invasion, margin status, and other clinico-pathologic parameters are essential to guide patient management and treatment.

## Deep generative models for image translation

The study of deep generative models (DGM) for imaging is a dynamic and active area within the AI research community. With the presentation of novel models and methodologies every year, generative image models have reached a level of maturity that has facilitated their widespread usage by the general public, with applications such as the AI art generators DALL-E^35^ or Stable Diffusion^36^.

In computational pathology, three key model architectures are commonly used for image-to-image translation tasks such as virtual staining. Generative adversarial networks (GANs)^10^ remain the most widely used model class, with architectures such as cycleGAN^11^ and conditional GAN (cGAN)^37^ being the most prevalent. Contrastive learning approaches, especially Contrastive unpaired translation (CUT)^38^, are also commonly employed, often in combination with GANs. Furthermore, diffusion models^39^, which have already demonstrated superiority over GANs in other fields, are gaining popularity for virtual staining tasks and are anticipated to gain wider adoption in the near future.

### Generative adversarial networks

**Fig. 2:**
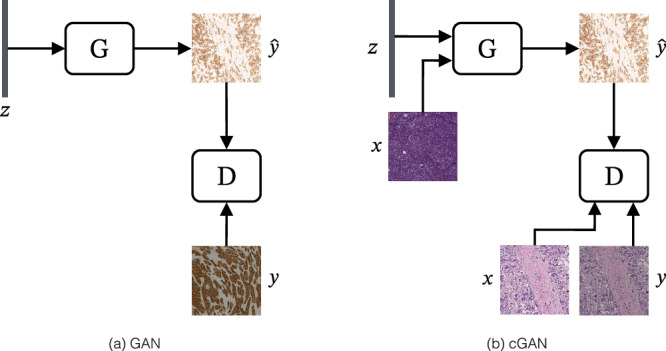
Generative adversarial networks (GANs) basic structures. **a** General GAN and (**b**) conditional GAN, where z is a noise vector, x is a real image from the source distribution, y is a real image from the target distribution, ${\hat{y}}$ is the generated image, D is the discriminator, and G is the generator. Example tiles adapted from the BCI dataset^62^.

GANs are a framework where two competing models are trained simultaneously (Fig. 2a). In the context of image generation, the first model (generator) tries to generate images that look real, while the second adversarial model (discriminator) tries to distinguish between the real training images and the generated images. Both models are trained by either minimising or maximising the training objective:

1 $$\mathop{\min }\limits_{G}\mathop{\max }\limits_{D}V(D,G)={{\mathbb{E}}}_{y \sim {p}_{{\rm{data}}}(y)}\left[\log D(y)\right]+{{\mathbb{E}}}_{z \sim {p}_{z}(z)}\left[\log \left(1-D(G(z))\right)\right]$$

where *D* is the discriminator, *G* the generator, *y* a real image from the target distribution, and *z* a noise vector.

This learning approach is analogous to a game between an art forger and an appraiser: the forger aims to produce perfect counterfeit artworks, while the expert attempts to distinguish between genuine and fake pieces. After each round, they receive feedback, allowing the forger to enhance their counterfeiting techniques and the appraiser to improve their detection strategies for later rounds.

While the original GAN takes noise as an input to the generator, it is also possible to use images instead. For virtual staining, where the goal is to transfer a source distribution (e.g., H&E images) to a target distribution (e.g., as IHC images), there are different ways to employ this framework for image-to-image translation, with the most common models being based on conditional GAN (cGAN)^37,40^ and cycleGAN^11^. The cGAN (Fig. 2b) extends the GAN framework by adding conditional information, such as the input image, *x*, to the training objective:

2 $$\mathop{\min }\limits_{G}\mathop{\max }\limits_{D}V(D,G)={{\mathbb{E}}}_{y,x \sim {p}_{{\rm{data}}}(y,x)}\left[\log D(y,x)\right]+{{\mathbb{E}}}_{z \sim {p}_{z}(z),x \sim {p}_{x}}\left[\log \left(1-D(G(z,x),x)\right)\right]$$

where, in the context of paired images (for example well registered H&E and IHC images from consecutive tissue sections) the conditional information could be, for example, the respective source H&E image, *x*, that is presented to the discriminator alongside the fake, *G*(*z*, *x*), and real, *y*, IHC images.

**Fig. 3:**
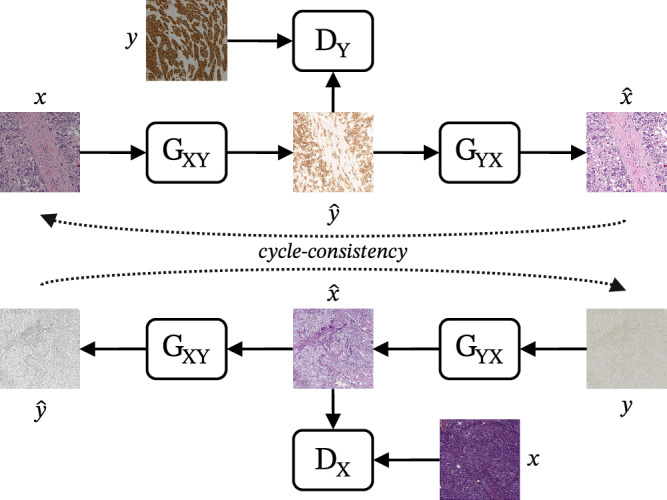
CycleGAN framework. *x* and *y* are real images from the source and target distributions, respectively, ${\hat{x}}$ and ${\hat{y}}$ are generated images, *G*_*XY*_ is the generator from the source to the target domain, *G*_*YX*_ the generator from the target to the source domain, *D*_*X*_ the discriminator for the source domain and *D*_*Y*_ the discriminator for the target domain. The cycle-consistency concept ensures that a generated image can be translated back to the source domain via the opposite generator. Example tiles adapted from the BCI dataset^62^.

However, medical imaging data often lacks proper pairings, and thus, we need a model architecture capable of matching unpaired data, such as the cycleGAN. In this case, we have two generators: *G*_*XY*_ that maps from the source to the target distribution (e.g., H&E to IHC) and *G*_*YX*_ that maps from the target to the source distribution (e.g., IHC to H&E). This framework employs the idea of cycle-consistency loss^11^, which ensures that an image from the source domain that has been translated by *G*_*XY*_ to the target domain and then translated back to the source domain via *G*_*YX*_ (from H&E to IHC and back to H&E), is almost identical the original input image, as depicted in Fig. 3. As intuition, similar to language translation, where it is expected that converting an English sentence to German and then back to English should result in the original sentence but since some meaning may be lost in translation, it is logical to enforce this consistency by including it into the training objective.

While cGANs and cycleGANs describe the high-level architecture, they consist of several distinct modules, such as the generator and the discriminator. The backbone of the generator is commonly a CNN-based model, such as a U-Net^41^ or ResNet^42^, which are usually pre-trained on existing image databases such as ImageNet^43^. The most common discriminator model is PatchGAN^40^, which divides the generated image into smaller patches (70 × 70 pixels in the original paper) and classifies each of them as either real or fake. This ensures that small local structures are sharp and works empirically better than classifying the whole image.

### Contrastive learning

**Fig. 4:**
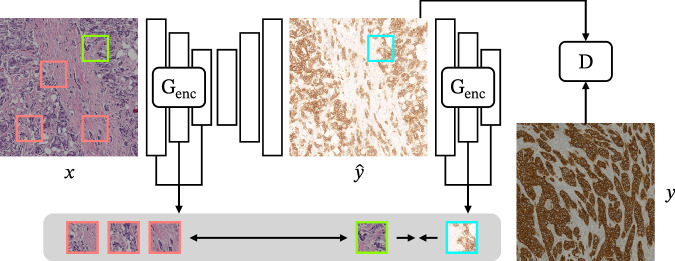
Contrastive unpaired translation (CUT) framework. *x* and *y* are real images from the source and target distributions, respectively, $\hat{y}$ is the generated image, *G*_enc_ is the generator encoder and *D* is the target discriminator. Example tiles adapted from the BCI dataset^62^.

Contrastive learning is a technique used to learn useful representations of an input by minimising the distance of embeddings of similar/matched inputs and maximising the distance between different/unmatched inputs. A common model for an image-to-image translation task is the contrastive unpaired translation (CUT) framework^38^, which compares a patch from the generated image (query) with its corresponding patch in the input image (positive) and other patches from the same input image (negatives). This is done by feeding both the input and the generated image to the generator encoder (*G*_enc_) and calculating the distance of the query, positive, and negatives in the different layers of the encoder. Maximising the distance between the query and the negatives while minimising the distance between the query and the corresponding positive patch leads to learning and preserving the content of the input image (Fig. 4). The loss function for this contrastive objective is called PatchNCE loss and can be combined with a standard adversarial GAN loss (balancing a generator and a discriminator) to generate images that have the style of the target domain but are close in content to the input image.

In the context of virtual staining, a further development of the PatchNCE loss is the adaptive supervised PatchNCE (ASP) loss. Instead of choosing positives and negatives from the input H&E image, Li et al.^44^ choose those embeddings from the ground-truth IHC image, on which they then calculate the supervised PatchNCE (SP) loss. Then, this loss is weighted by a cosine similarity of the embeddings of each anchor and positive patch in the generated and real IHC, resulting in the ASP loss. With this strategy, regions with low consistency between each other have less impact on training. It is worth mentioning that this loss is only introduced at later stages of model training when a proper distinction between consistent and inconsistent patch locations can be made.

### Diffusion models

**Fig. 5:**
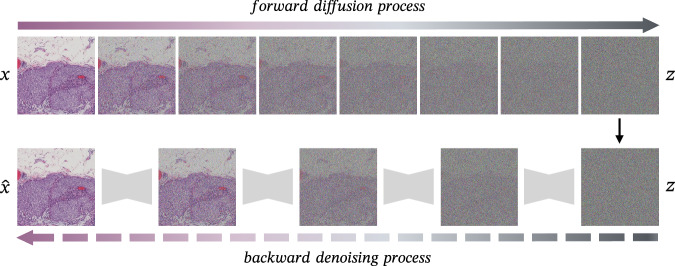
Diffusion models’ basic concept. The diffusion process iteratively adds noise (z), while the denoising process aims to reconstruct the original image (x). Example tile adapted from the BCI dataset^62^.

Diffusion models (Fig. 5) are a class of latent variable models^45^ that are inspired by diffusion processes in thermodynamics^46^. In such a model, an input image (*x*) is slowly corrupted by iteratively adding small amounts of Gaussian noise (*z*), a process which is called the forward/diffusion process. Then, during the backwards/denoising process, a neural network gradually denoises the image until it reconstructs the original input image. This process is done by predicting the noise that was added at a certain time step (with a U-Net, for example) and then subtracting it from the image at that time step. Using a trained denoising network, it is now possible to generate images sampled from a noise distribution. This network can also be conditioned on modalities such as text (e.g., “generate an image that shows tumour tissue”) or images (e.g., the H&E source image). In addition to generating images from noise, such models can also be employed for tasks such as super-resolution^47^ or style transfer^48^. Recently, diffusion models have gained popularity due to their stability and easier training schemes when compared to GANs, while still resulting in good image quality^49,50^.

## Virtual staining evaluation

Assessing the performance of virtual staining methods requires appropriate evaluation metrics. Since different metrics highlight different aspects of performance, it is crucial to select appropriate ones for a fair and comprehensive assessment. The following section will provide an overview of some commonly used evaluation metrics for virtual staining. Although virtual staining is, in essence, an image-to-image translation task, and thus we can use well-established metrics in the field of computer vision, it also has some unique requirements specific to pathology. While in a natural image, the colour of an apple in a fruit basket does not matter as long as it looks like an apple, in histopathology, it matters if a specific cell is stained or not. Many methods try to define pathology-related metrics and loss functions (that also consider imperfect image registration) to reach increased pathological consistency with the ground-truth. Although quantitative metrics help in elucidating the performance of a model, in the end, the goal is to use virtual staining networks in the clinics. Some authors, therefore, try to assess how Pathologists rate the resulting generated images and whether they can distinguish them from real images.

### Standard image-to-image metrics

One of the simplest ways to evaluate if two images are alike is to compare them pixel by pixel. We can subtract the pixel values of a generated image $\hat{y}$ from the pixel values of the ground-truth image *y* and sum up the absolute values to see how similar both images are. This sum is called L1 (Eq. (3)), and if divided by the total number of pixels, n, it becomes the mean absolute error (MAE), defined in Eq. (5). The second related measure is L2, also called Euclidean distance, which is defined as the square root of the sum of the squared differences between pixels, as in Eq. (4). If averaged over all squared distances, we get the mean squared error (MSE), as in Eq. (6). L1 and L2 are common loss terms in the objective functions for image-to-image translation models, but tend to lead to blurry generated images when solely relying on them.

3 $${L}_{1}=\mathop{\sum }\limits_{i=1}^{n}| {y}_{i}-{\hat{y}}_{i}|$$

4 $${L}_{2}=\sqrt{\mathop{\sum }\limits_{i=1}^{n}{({y}_{i}-{\hat{y}}_{i})}^{2}}$$

5 $$\,{\text{MAE}}\,=\frac{1}{n}\mathop{\sum }\limits_{i=1}^{n}| {y}_{i}-{\hat{y}}_{i}|$$

6 $$\,{\text{MSE}}\,=\frac{1}{n}\mathop{\sum }\limits_{i=1}^{n}{({y}_{i}-{\hat{y}}_{i})}^{2}$$

A measure that takes the MSE into account is the peak signal-to-noise ratio (PSNR), Eq. (7), where MAX is the maximum value a pixel can attain (e.g., 255 in an 8-bit image). PSNR is expressed in decibels, and higher values (going towards infinity when approaching an MSE of 0) indicate better correspondence:

7 $$\,\text{PSNR}\,=10\cdot {\log }_{10}\left(\frac{{\text{MAX}}^{2}}{\text{MSE}\,}\right)$$

In virtual staining, using MAE, MSE, or PSNR to compare model performance is not ideal, as, in stain-to-stain translation scenarios, ground-truth and source images usually do not have good pixel-to-pixel correspondence. This can skew the metrics, and thus, they have been found to be poorly aligned with the human perception of image similarity. The structural similarity index (SSIM)^51^ tries to alleviate this problem and represents a measure that takes into account the luminance (*l*), contrast (*c*), and structure (*s*) of both images, as in Equation (8):

8 $$\,{\text{SSIM}}\,(y,{\hat{y}})={\left[l(y,{\hat{y}})\right]}^{\alpha }\cdot {\left[c(y,{\hat{y}})\right]}^{\beta }\cdot {\left[s(y,{\hat{y}})\right]}^{\gamma }$$

9 $$l(y,\hat{y})=\frac{2{\mu }_{y}{\mu }_{\hat{y}}+{C}_{1}}{{\mu }_{y}^{2}+{\mu }_{\hat{y}}^{2}+{C}_{1}}$$

10 $$c(y,\hat{y})=\frac{2{\sigma }_{y}{\sigma }_{\hat{y}}+{C}_{2}}{{\sigma }_{y}^{2}+{\sigma }_{\hat{y}}^{2}+{C}_{2}}$$

11 $$s(y,\hat{y})=\frac{{\sigma }_{y\hat{y}}+{C}_{3}}{{\sigma }_{y}{\sigma }_{\hat{y}}+{C}_{3}}$$

where (*μ*_*y*_) and ${\mu }_{\hat{y}}$) are the mean intensities, *σ*_*y*_ and ${\sigma }_{\hat{y}}$ are the standard deviations, ${\sigma }_{y\hat{y}}$ is the covariance, *α*, *β*, and *γ* determine the weighting of each component, and *C*_1_, *C*_2_, *C*_3_ are constants added to avoid instability. The SSIM is more robust than the aforementioned metrics to small distortions and generally shows higher concordance with human perception. The multi-scale SSIM (MS-SSIM)^52^ constitutes an extension of the SSIM index, combining measurements across multiple scales of the image, thereby offering greater robustness to distortions and variations in viewing conditions.

Although straightforward, the PSNR and SSIM metrics are limited in their ability to capture the subtleties of human perception fully. Zhang et al.^53^ demonstrated that networks trained for visual prediction tasks inherently learn representations of the world that correlate strongly with human perceptual judgement. Notably, the more effective a feature set is at tasks such as classification, the more robust it becomes as a model of perceptual similarity. This insight underpins the development of several perceptual similarity metrics, including the Learned Perceptual Image Patch Similarity (LPIPS), which quantifies image similarity by measuring the distance between activations in a network trained on human similarity judgements. The metric between two paired images (*x*_1_ and *x*_2_) is calculated by computing the L2 distance of channel-normalised activations from different layers (${\hat{y}}_{1hw}^{l}$ and ${\hat{y}}_{2hw}^{l}$), multiplying them by a weight vector, averaging them spatially, and summing them channel-wise:

12 $$d({x}_{1},{x}_{2})=\sum _{l}\frac{1}{{H}_{l}{W}_{l}}\sum _{h,w}{\left\Vert {w}_{l}\odot \left({\hat{y}}_{1hw}^{l}-{\hat{y}}_{2hw}^{l}\right)\right\Vert }_{2}^{2}$$

A metric specifically introduced to measure the performance of GANs is the Fréchet Inception Distance (FID)^54^, which measures the Wasserstein distance between the embeddings of the generated and real image distributions, under the assumption that both follow a Gaussian distribution. Thus, this is a metric that highlights how well the model has captured the real distribution and not just pairwise concordance. First, embeddings for a set of generated and real images are generated using the Inception v3^55^ model. Then the score is given by calculating the Euclidean distance between the means of the feature vectors (*μ*_*y*_ and ${\mu }_{\hat{y}}$) of both sets and adding the trace (Tr) of a combination of the covariance matrices (Σ_*y*_ and ${{\rm{\Sigma }}}_{\hat{y}}$) to it, Eq. (13). The more similar both distributions are, for example, the more similar the generated images are to the real images in the embedding space of the Inception model, the smaller the FID score will be. This score represents the state-of-the-art in evaluating image-to-image models and can be used for unpaired datasets and unsupervised methods. It must be noted that this metric is affected by the number of images which make up both distributions, and thus, when comparing models, this should be taken into account^56^.

13 $$\,{\text{FID}}\,(y,{\hat{y}})={\left\Vert {\mu }_{y}-{\mu }_{\hat{y}}\right\Vert }_{2}^{2}+\,{\text{Tr}}\,\left({{{\Sigma }}}_{y}+{{{\Sigma }}}_{\hat{y}}-2{\left({{{\Sigma }}}_{y}{{{\Sigma }}}_{\hat{y}}\right)}^{1/2}\right)$$

### Pathology-related metrics

While models that perform well on image-to-image metrics are valuable, they must also preserve and highlight pathological information that is present in the slides. As the chromogen concentration in an image is linked to the protein expression of the biomarker of interest, some authors focus on metrics that involve this channel by mapping the RGB to the HED (haematoxylin, eosin, and DAB) colour space^57^. Since the DAB channel represents the chromogen concentration, a simple distance measure, like Jensen-Shannon, between the intensity distributions of real and generated channels can be meaningful. Another option is to compare the number of positively stained cells in the real versus the generated image based on some intensity threshold, as done by Martino et al.^58^. Moreover, a threshold (given by the clinical guidelines defined in Fig. 1) defining whether an image is positive or negative based on the percentage of stained cells allows for an assessment of concordance with the ground-truth. The DAB channel can even be used to define segmentation maps for training or evaluation.

Another approach to evaluate a virtual staining model is to use its output for downstream tasks such as the classification of biomarker status. For example, Peng et al.^59^ trained a classifier based on the generated and the real IHC to predict HER2 status and compared its performance. This is relevant as the primary focus in clinical practice is not necessarily the appearance of the image itself, but rather the insights it provides about the underlying biomarker status, crucial to drive clinical decision-making. While the assessment of downstream tasks is useful and important for evaluating the potential applicability in practice, most methods do not provide such evaluations, which might also be partly due to insufficient virtual staining quality across many methods.

### Qualitative evaluations

To evaluate the usability of the developed models, some researchers present the generated images to pathologists for a qualitative evaluation that typically assesses whether pathologists (1) can distinguish between generated and real IHC images and (2) draw the same conclusions, such as the biomarker status, based on the generated slides. For example, Zeng et al.^60^ showed PgR positive and PgR negative slides to a Pathologist to determine the concordance of positivity status of real with generated slides. In another instance, Martino et al.^58^ showed 30 synthetic IHC images next to the real IHC images to 2 pathologists and let them decide if the image was generated or real.

## Pathology datasets for breast IHC biomarkers

A deep learning-based model can only be as good as the data on which it was trained, and thus, high-quality datasets are important. Among the criteria that need to be considered when choosing a dataset to train virtual staining models are: (1) the quality of image registration, (2) the prevalence of artefacts and (3) the balance of samples with different staining levels. Deep learning based models can easily overfit and produce great results for the dataset they are trained on, but then fail to translate this performance to out-of-distribution data, such as datasets with different staining profiles. Thus, it is important to use data from different sources (e.g., different labs), as well as apply best practices accounting for domain generalisation during training, such as colour normalisation and data augmentation^61^ which can mitigate staining differences due to scanner types, staining protocols, and other factors that vary between labs, or even in the processing of samples within the same dataset.

Specifically to train virtual staining models for breast cancer biomarkers, there are some publicly available datasets (summarised in Table 1) that can be used, as they include both H&E and IHC images of the same tissue sample (consecutive cuts). The most notable, as they are the most accessible and widely used, are the breast cancer immunohistochemical (BCI) dataset^62^ and the Multi-IHC Stain translation (MIST)^44^ dataset. The BCI dataset comprises 4873 pairs of well-registered 1024 × 1024 px tiles of consecutive H&E and HER2 IHC-stained whole-slide images (WSIs) from 51 patients. The WSIs were initially manually aligned using projection transformation and further registered with the Elastix toolbox^63^. As one of the first publicly available datasets of paired H&E and IHC slides, it is widely used in the field. The MIST dataset also contains paired consecutive 1024 × 1024 px tiles of H&E, HER2 (*n* = 5, 642 from 64 WSI), ER (*n* = 5, 153 from 56 WSI), PgR (*n* = 5, 139 from 56 WSI), and Ki67 (*n* = 5, 361 from 56 WSIs) stainings. Another collection that includes registered tiles but that has not been used for virtual staining so far is the IHC4BC dataset^64^. It contains images from 231 WSI pairs, with a total of 92,769 patch pairs, and includes stainings for HER2, ER, PgR and Ki67.

**Table 1:** Summary of the publicly available datasets with image pairs of H&E and (at least one of) the breast cancer IHC biomarker(s)

Data	Name	IHC	# of images	Registered	Resolution (mpp)	Scanner
Tiles	BCI	HER2	9746	Yes	0.46 (20×)	Hamamatsu NanoZoomer S60
	MIST	Breast panel	21,295	Yes	0.46 (20×)	KFBIO KF-PRO-005
	IHC4BC	Breast panel	185,538	Yes	0.26 (40×)	Leica Aperio GT 450
Slides	HER2 Contest	HER2	172	No	0.23 (40×)	Hamamatsu NanoZoomer C9600
	AIDPATH	Breast panel	500	No	0.25 (40×)	Leica Aperio CS
	ACROBAT	Breast panel	4212	No	0.92 (10×)	Hamamatsu NanoZoomer XR/S360
	ANHIR	HER2, ER, PgR	90	No	0.25 (40×)	Leica Aperio AT2

In contrast to the aforementioned three datasets, several others contain pairs of H&E and IHC stained slides. The HER2 contest^65^ dataset contains 172 IHC and H&E WSIs from 86 cases of invasive breast carcinomas. The AIDPATH dataset^66,67^ contains a total of 500 slides of H&E, HER2, ER, PgR and Ki67 stains, from 50 patients. The ACROBAT data collection^68^ is the largest publicly available WSI image dataset (*n* = 4, 212) of BC patients, including H&E, HER2, ER, PgR and Ki67 stained WSIs from 1153 patients. The ANHIR dataset^69^ is part of a challenge to improve the registration of H&E to IHC WSIs, which includes a subset of 30 pairs of breast tissue samples, including HER2, ER, and PgR stains.

## Advancements in virtual staining for breast IHC biomarkers

**Fig. 6:**
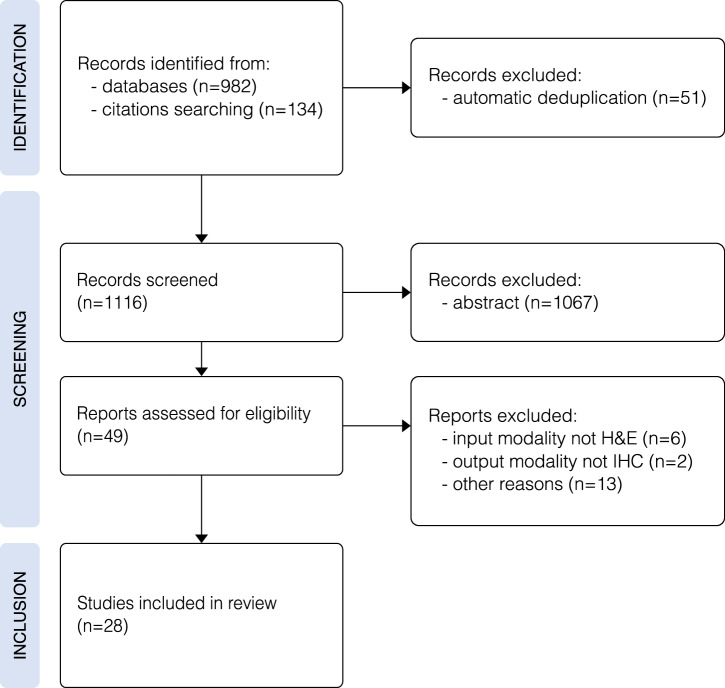
PRISMA flowchart illustrating the selection process for the studies included in the review, identified via databases and other sources.

We have identified 28 works published since 2021 that were developed for virtual staining (H&E to IHC) of the clinical biomarker panel for breast cancer described in Section “Breast cancer and clinical IHC biomarkers”. This list is the result of a bibliographic search of Google Scholar using the keywords ("virtual staining” or “virtual histological staining” or “computational staining” or “digital staining” or “synthetic staining”) and ("HER2” or “PR” or “PgR” or “ER” or “KI67”), checking the citations of key papers, as well as manually going through publications of relevant conferences and authors with previous work in the field (Fig. 6). Overall, this process resulted in 1116 potential articles that were assessed and subsequently rigorously analysed, resulting in the inclusion of 28 relevant works, all summarised in Table 2. These publications can be categorised into different model architectures, for example, whether they employ a GAN or a Diffusion model framework. We decided to further differentiate whether a model used some form of contrastive learning. While these models usually use a contrastive learning objective together with a GAN, we believe that they are distinct enough to group them.

**Table 2:** Summary of the state-of-the-art models on virtual staining (H&E to IHC) of the clinical biomarker panel for breast cancer

Framework	Author/model	Year	HER2	ER	PgR	Ki67	Datasets	Details	Q.E.
GANs	Liu et al.^70^ PC-StainGAN	2021	-	-	-	x	In-house: breast + neuro	CycleGAN that predicts tumour area heat maps and aligns the latent space between both generators.	-
	Zeng et al.^60^	2022	-	-	x	-	In-house: breast	CycleGAN that uses a positive/negative biomarker classifier and class activation maps to focus the model on certain regions.	x
	Liu et al.^62^ pyramid Pix2Pix	2022	x	-	-	-	BCI	Conditional Pix2Pix with additional pyramidal loss (L1 loss on the ground-truth at different scales after downsampling and blurring) and a discriminator based on PatchGAN.	x
	Duan et al.^110^	2023	x	-	-	-	BCI	A generator consisting of a style and a content encoder and a discriminator that uses an additional biomarker classifier.	-
	Ma et al.^75^ CAS-Transformer	2023	x	-	-	-	BCI	Swin-Transformer backbone and pre-training phase based on masked image modelling with fine-tuning using a GAN with a PatchGAN discriminator.	-
	Huang et al.^111^ TC-cycleGAN	2023	x	-	-	-	BCI	CycleGAN that includes a loss function based on Gabor filters.	-
	Baldeon-C. et al.^81^ DeepSIT	2023	x	-	-	-	BCI	U-Net generator with four PatchGAN discriminators on the outputs of different depths of the U-Net decoder.	-
	Liu et al.^112^ MGGAN	2023	x	-	-	-	BCI	GAN with a generator consisting of 2 U-Nets that extract features at different scales (3 × 3 or 7 × 7 kernels in the first convolutional layer).	-
	Martino et al.^58^	2024	-	-	-	x	In-house: oral	Standard Pix2Pix model without any added loss functions.	x
	Ma et al.^84^ DSFF-GAN	2024	x	x	-	x	MIST In-house: endometrial	CycleGAN that generates positive region masks, which are compared against masks based on the DAB channel of the ground-truth image. Additionally, a latent space loss is added.	x
	Wu et al.^113^ HcGAN	2024	x	x	x	x	BCI MIST	Conditional GAN with a U-Net generator and harmonic convolutions based on discrete cosine transforms.	-
GANs	Peng et al.^59^ TDKstain	2024	x	-	-	-	In-house: breast	Conditional GAN with a regularised training based on generated/real nuclei density maps and L1 loss on the generated DAB channel.	-
	Wei et al.^86^ DeReStainer	2024	x	-	-	-	BCI	A GAN model that processes the shared haematoxylin channels of the H&E and IHC images to constrain the training and uses a HER2-classifier to regularise the decoder.	-
	Guan et al.^88^ GramGAN	2024	-	-	-	x	In-house: kidney + lung	A multi-domain stain-transfer model based on cycleGAN, using a style-encoding dictionary and auxiliary stain-type classifiers for regularisation.	-
	Qu et al.^83^ BCIStainer	2024	x	-	-	-	BCI	A GAN with parameter-free attention layers in the generator and two classifiers to predict HER2-expression levels on both the H&E and IHC images.	-
Contrastive learning	Zhang et al.^89^ MVFStain	2022	x	x	x	x	In-house: lung, heart, vascular, breast	A multi-domain stain transfer GAN-based model, with style and content encoders, that uses a histogram loss to separate feature distributions of different stains.	x
	Li et al.^44^ ASP	2023	x	x	x	x	BCI MIST	Model based on pyramid Pix2Pix and CUT, which includes a supervised contrastive learning loss weighted by the correspondence between the generated image and the ground-truth.	-
	Qu et al.^91^	2024	x	-	-	-	MIST	Model based on ASP, using multiple scales of the input and attention modules as skip-connections.	-
	Chen et al.^93^ PSPStain	2024	x	-	-	-	BCI MIST	ASP-based model that integrates DAB-channel losses and cross-image probability maps based on the tumour region to increase pathological information consistency.	-
	Li et al.^94^ WPCC	2024	x	x	x	x	BCI MIST	CUT-based model that calculates the Jensen-Shannon divergence (JSD) between the distributions of contrastive losses calculated on the real and the generated image.	-
	Wang et al.^95^ MDCL	2024	x	x	x	x	BCI MIST	GAN framework with two siamese networks with the generated IHC and H&E (or real IHC) as input. Corresponding patch embeddings of H&E, real and generated IHC are pulled together while spatially non-corresponding patches are pushed apart.	-
	Zhang et al.^96^ PPT	2024	x	-	-	-	BCI MIST	GAN that expands the PatchNCE loss by using the focal loss on all three combinations of real/fake IHC and the H&E input.	x
	Li et al.^98^ Confusion-GAN	2024	x	x	-	-	BCI MIST In-house: liver	A cycleGAN with a multi-branch generator and discriminator to handle features at multiple scales, an auxiliary classifier based on stained-region percentage to capture pathological information and a contrastive loss to between positive/negative real/generated patches.	x
	Hu et al.^114^ ULViT-GAN	2024	x	-	-	-	BCI	Based on cycleGAN, the generator has a U-Net-like structure with additional Harr-wavlet downsampling and a vision transformer module on the output of the encoder.	-
Diffusion models	He et al.^101^ PST-DIFF	2024	x	x	-	x	BCI ACROBAT	Two diffusion models that learn to denoise their respective domains. The latent space is connected via the dual diffusion implicit bridge method, and structural consistency is learned in the de-noising model via conditioning on frequency information.	-
	Li et al.^104^ His-MMDM	2024	-	-	x	x	In-house: brain	A diffusion model conditioned on source and target domains that can also be conditioned on mutations or tissue type.	-
Other models	Jia et al.^115^ DTNet	2024	x	x	x	x	In-house: breast	U-Net architecture with content and style encoders, transformer skip connections and a pyramidal loss.	-
	Ji et al.^116^	2024	-	-	-	x	In-house: uterus	A U-Net with an H&E image converted to optical density as input.	-

*Q.E.* qualitative evaluation.

### Generative artificial networks

The first published study on virtual staining of a breast cancer IHC biomarker (Ki67) from H&E-stained samples was developed by Liu et al.^70^ in 2021, based on the cycleGAN framework. The authors argued that cycleGAN lacks constraints between the generated and target images, which may lead to inconsistencies in structural and pathological content during image translation. To address this limitation, they introduced a pathological representation network (PRN) within each generator. This network predicts areas of tumour cells, constraining the model by penalising deviations between the predicted and ground-truth heatmaps, as well as discrepancies between predicted heatmaps across generators, using the L1 loss. Additionally, the authors use an SSIM-cycle loss and also incorporate a base-space loss to align the latent space of both generators. This work was developed on in-house datasets of neuroendocrine and breast tissue, with the last comprising 160 pairs of H&E and Ki67-stained WSIs, divided into 288 × 288 pixel tiles (resulting in 57,000 tiles per pair). In comparison with cycleGAN, the proposed model (PC-StainGAN) presents better MAE, SSIM, MS-SSIM, and PSNR between the H&E source and reconstructed images, better Contrast Structure Similarity (CSS, a metric based on the SSIM) between the H&E source and generated IHC images, and a better Pearson correlation between the positive- and negatively-stained areas of the generated and reference IHC images.

In 2022, Zeng et al.^60^ devised a semi-supervised method for virtual staining of PgR in breast tissue. After registering^71^ the consecutive H&E and IHC slides and generating the corresponding paired tiles, a positive/negative label is assigned to each pair based on DAB channel thresholding. Thus, the cycleGAN architecture can be complemented with a positive/negative classification loss, constraining the input and generated images to retain consistency with their labels. In addition, the authors added an auxiliary classification loss (CAM loss^72,73^) to focus on more discriminative areas (attention map). The proposed framework was trained on an in-house BC dataset consisting of 22 training pairs of slides (58,942 and 62,218 of H&E and PgR overlapping 256 × 256 px patches, respectively) and 8 different pairs for evaluation, achieving better image metrics when compared to cycleGAN, MUNIT^74^ or UGATIT^73^ models, while also showing high agreement with the biomarker status, when evaluated by a pathologist.

In addition to publishing the BCI dataset, Liu et al.^62^ also improved the classic Pix2Pix model^40^. Since perfect pixel alignment is not possible for consecutive tissue samples, the authors proposed a pyramid framework that applies Gaussian kernels and downsampling to both the generated and ground-truth images, thus ensuring better consistency between pairs at multiple scales. The standard GAN and L1 loss of the vanilla Pix2Pix generator are then joined by a multi-scale L1 loss applied to the downsampled and smoothed image pairs. Using ResNet9-blocks as the generator backbone and PatchGAN as the discriminator, they report higher PSNR and SSIM than other Pix2Pix models. This paper represents a significant milestone in virtual staining for BC immuno-biomarkers, as the BCI dataset is widely used, and the pyramid Pix2Pix model is a base framework for many subsequent models. However, when assessed by two pathologists, less than 40% of the generated images were evaluated with the same HER2 expression as the corresponding real image, showing that there is still a lot of room for improvement before these models can be deployed for clinical use.

While most GAN frameworks use a CNN-based model as the building block, in 2023, Ma et al.^75^ employed the Swin-Transformer^76^ as the model backbone, to balance local and global feature learning, and a pre-training method based on masked image modelling (MIM)^77,78^, to enhance the model’s performance and ensure it learns meaningful representations from a labelled dataset. The pre-training phase was done in the Insillico Labelling dataset (ISL)^79^, which includes 148,454 pairs of un-stained/stained microscopy images with 256 × 256 px, before a fine-tuning phase on the BCI dataset. In the fine-tuning phase, the authors used the PatchGAN^40^ discriminator and the Charbonier loss function^80^ (a smoothed version of MAE loss), achieving the highest PSNR and second-highest SSIM values when compared to Pix2Pix, pyramid Pix2Pix and cycleGAN frameworks.

Baldeon-Calisto et al.^81^ trained a U-Net generator on the BCI dataset, employing four PatchGAN discriminators at different depths of the U-Net decoder. The training process was constrained by a combination of GAN and L1 losses, computed between the generated outputs at different depths and the ground-truth. Intermediate outputs were resized via bilinear interpolation to align with the dimensions of the real IHC images. The authors reported a superior SSIM value compared to other models, including Pix2Pix, Pix2PixHD^82^, pyramid Pix2Pix and cycleGAN. Notably, their model secured the top position in the post-challenge phase leaderboard of the BCI GrandChallenge^83^. Furthermore, a qualitative evaluation of the generated outputs demonstrated optimal performance on 0 and 1+ HER2 scores but diminished performance on 3+ HER2 scores, consistent with prior studies’ observations.

In 2024, based on PC-StainGAN, Ma et al.^84^ avoid the need for expert annotation segmentation masks by converting an IHC-stained RGB image to the HED colour space and then thresholding the DAB channel. They also introduce the Dual Scale Feature Fusion (DSFF) block in the generator’s decoder, which combines upsampling with Pathological Feature Attention (PFA) blocks. The PFA sub-block enhances the network’s colouring ability by calculating attention weights for the channel, width, and height dimensions. Additionally, they modified the SSIM-loss to account for the colour fidelity of virtually stained images, penalising differences between the pixel values in the XYZ colour space. The authors trained and evaluated their model on an in-house dataset of Ki67-stained endometrial samples. Their experiments demonstrated superior SSIM, PSNR and CSS compared to variations of the pyramid Pix2Pix and cycleGAN models. For Ki67, the authors also had two Pathologists quantifying the positive cells ratio per patch, and then compared against the ratio on the corresponding real patches. Their model achieved the smallest deviation and lowest variation among the evaluated models. Additional testing on the MIST dataset confirmed the model’s effectiveness on SSIM and CSS for ER and HER2 stains.

With the same goal of ensuring accurate pathological representation without expert annotations, Peng et al.^59^ proposed the TDKstain model, leveraging task-specific domain knowledge by extracting the stained membrane regions from the DAB channel and nuclei density maps from the haematoxylin channel. A GAN model is then coupled with a density estimation network, with both networks trained alternately, to generate the IHC staining and predict the DAB channel from the H&E input and its corresponding membrane staining intensity mask. The authors used an in-house dataset of breast tissue comprising well-registered H&E and HER2-stained non-overlapping images, each with a fixed size of 1024 × 1024 pixels. The model achieved the best values for FID and Deep Image Structure and Texture Similarity (DISTS)^85^ among SOTA models, although it did not achieve the highest SSIM score. Moreover, the model excelled in task-specific domain evaluations, including nuclei density estimation and membrane staining intensity. In contrast to most of the other works, the authors tested their method on a downstream task, reporting similar performance for a HER2-grade classification model trained on generated IHC-stained images and the same model trained on real IHC-stained images.

Wei et al.^86^ utilise the shared haematoxylin (H) channel between H&E- and IHC-stained images. Their generator includes a DeStainer encoder, which extracts and encodes the haematoxylin channel, and a symmetric ReStainer decoder that applies the virtual DAB stain to generate the IHC-stained image. During training, both the H&E and IHC haematoxylin channels are fed to the DeStainer encoder. After encoding, the inputs are aligned, and HER2 grade is estimated from the H&E haematoxylin channel. The alignment task uses a cosine similarity loss, and HER2-grade estimation is constrained by Focal loss^87^. The ReStainer then predicts the DAB channel, constrained by L1-loss between the virtual and ground-truth IHC DAB channels. A comparator module assesses the similarity between the virtual and real IHC images using a combination of SSIM, MAE, and cosine similarity. In evaluations on the BCI benchmark, the model achieved top SSIM results but did not achieve state-of-the-art performance on PSNR. Additionally, the authors also trained a k-Nearest Neighbours (kNN) classifier on the generated images, and while accuracy is not sufficient (60%), the model trained on their images outperforms the ones trained on images generated by ASP and pyramid Pix2Pix.

Guan et al.^88^ proposed a multi-domain stain transfer method (GramGAN) that enables transfer across multiple staining styles using a single network. Based on cycleGAN^11^, this model introduces style-guided (SG) blocks in the generator’s bottleneck to progressively transfer the stain from the original to the target by leveraging a style encoding dictionary (SED) to adjust the input feature map towards the target style incrementally. The discriminator architecture includes an auxiliary classifier, an auxiliary discriminator and a target stain classifier. After passing through the downsampling module, the auxiliary classifier and discriminator generate attention weights that guide the respective components. The authors trained and evaluated GramGAN on three datasets with different markers, including Ki67 data from the ANHIR^69^ dataset, corresponding to 8961 H&E tiles and 8419 Ki67 tiles from three lung tumour samples. Their model outperformed several SOTA models in CSS, FID, and kernel inception distance (KID) metrics, as well as in a downstream task of glomeruli segmentation and detection.

### Contrastive learning

In 2022, Zhang et al.^89^ proposed the first network to generate multiple IHC stains from H&E, including HER2, PgR, ER, and Ki67. The multiple virtual functional stain (MVFStain) model generator consists of style and content encoders, as well as one decoder that merges the source/target content and style into a generated image. The content encoder uses pixel/channel-wise attention, while the style encoder constrains the feature distributions to a Gaussian distribution using KL loss. The decoder outputs three images: (a) a reconstruction of the source image, (b) the virtually stained image, and (c) a virtually stained image with a Gaussian-style feature vector as input. Images a and c are used for the reconstruction and cycle losses, respectively, and images b and c serve as input for a style discriminator. Additionally, a histogram-based loss, based on Ustinova et al.^90^, is applied to distinguish style feature distributions of inter- and intra-domain pairs. A second discriminator is applied to the content features. The authors used four datasets with different tissue types and staining, including ER, PgR, and HER2 for breast (in-house) samples. For this application, they used 5242 tiles of 256 × 256 px extracted from WSI from 3 individuals. The reported results showed higher PSNR and HTI for HER2 compared to a vanilla cycleGAN, as well as better PSNR but slightly lower HTI for ER and PgR. Additionally, the average optical density (mIOD)^89^ for HER2 was closer to ground-truth in cycleGAN, while MVFStain performed better on ER and PgR. Moreover, two pathologists (blindly) graded both virtually- and manually-stained tissue sections on a scale of 1–3 for stain quality, with results showing that, overall, virtual images were nearly equal to the real ones.

In 2023, the landmark publication from Li et al.^44^ not only released the multi-IHC stain translation (MIST) dataset but also introduced the Adaptive Supervised PatchNCE (ASP) loss, built upon the work of Liu et al.^62^ and Park et al.^38^. When validating on MIST and BCI datasets, the authors report superior FID, KID, and Perceptual Hash Value (PHV) metrics compared to Pix2Pix^40^, cycleGAN and a CUT^38^ model with additional multi-scale L1 loss.

In 2024, Qu et al.^91^ introduced a model that uses a multi-magnification processing strategy and an attention module. After downsampling the original images, they randomly cropped and zoomed in on regions, resulting in images with 2× or 4× increased magnification. Using a super-resolution model^92^, the processed images were then resized to the same shape. This preprocessing of the input images was intended to ensure that the model is exposed to and learns to extract features from multiple scales. The general structure of the model follows the architecture of the ASP model with the addition of the attention module. The attention module connects both downsampling and upsampling blocks on the same level, as well as concatenates the output of the preceding layer. Thus, it not only focuses the module on certain spatial regions and feature channels but also functions as a skip connection. The evaluation of the model on MIST showed slightly better performance than the baseline ASP^44^ model.

Chen et al.^93^ extended the work of Park et al.^38^ and Liu et al.^62^ by addressing the issues of incorrect spatial alignment and the preservation of pathologically relevant information with their pathological semantics-preserving learning (PSPStain) framework. PSPStain employs two core strategies: the protein-aware learning strategy (PALS) and the prototype-consistent learning strategy (PCLS). In PALS, Focal Optical Density (FOD) maps are derived by isolating the DAB channel. Three DAB-specific losses are then calculated to retain biomarker expression using the FOD maps of both the real and generated IHC images: average difference, histogram difference, and average differences across 16 smaller patches. In PCLS, the authors use the output from the penultimate layer of a pre-trained UNet, along with class-label probability maps, to create prototypes for both real and generated IHC images. They then compute the cosine similarity between the generated IHC feature maps and the ground-truth IHC prototypes to derive a cross-image probability prediction. This prediction is compared to the respective generated mask, resulting in a cross-image tumour prototype consistency (CTPC) loss. Using both the BCI and MIST datasets, despite having lower scores on classic image quality metrics such as PSNR and SSIM when compared to CUT^38^, ASP, Pi2xPix and pyramid Pix2Pix models, the proposed model achieved top results in pathology-related metrics such as cumulative optical density (IOD), mIOD and the Pearson correlation coefficient.

Constructing upon the work of Park et al.^38^, Li et al.^94^ addresses the problem of the scarcity of pixel-wise paired datasets by introducing weakly pathological consistency constraint (WPCC) acting on multiple layers of the generator. WPCC starts with the extraction of the embedding vectors of both real and generated IHC images at various layers. Subsequently, it computes the distribution of patch embedding similarities in each layer and calculates the Jensen-Shannon divergence (JSD) between the real and generated distributions. Finally, using the Pearson correlation coefficient, the degree of alignment between consecutive H&E and IHC image tiles is determined. This alignment is then used to perform a weighted sum of the previously computed distances, with a higher degree of alignment corresponding to a larger weight. In addition to WPCC, the authors also introduce a discriminator regularisation loss function to stabilise the discriminator’s training. The model was trained on the BCI dataset, achieving better PSNR, FID, KID and SSIM when compared to cycleGAN, CUT, pyramid Pix2Pix and ASP.

Wang et al.^95^ propose mix-domain contrastive learning (MDCL), which, by employing two Siamese networks, estimates the correlation between the anchor and both inter- and intra-domain patches. For that purpose, upon IHC generation, some patches are randomly selected from the virtual IHC and input H&E to construct the anchor and positive sets, respectively, which will serve as the input for one of the Siamese networks. The other network’s input corresponds to the virtual IHC anchor set and the corresponding patches from the ground-truth IHC. However, since these are not pixel-level matched, an adaptive weighting factor is taken into consideration^44^. The contrastive loss function consists of a modified version of the one employed by Park et al.^38^ to measure the similarity in both the inter- and intra-domain. The authors trained and tested their model in the MIST^44^ and BCI^62^ datasets and demonstrated its superior performance on FID, KID, and PHV metrics when compared to cycleGAN, CUT, Pix2Pix, pyramid Pix2Pix and ASP.

Zhang et al.^96^ introduces the Patch alignment-based Paired (PPT) model, which employs bidirectional contrastive learning to alleviate the misalignment problem. Building upon the work of Li et al.^44^, the authors employ FocalNCE loss between the input and output images, as well as between the output and ground-truth images in a bidirectional manner. The FocalNCE loss consists of applying Focal loss^87^ to alleviate class imbalance over the PatchNCE loss function. In addition, the model’s training is constrained by consistency loss, which comprises patch loss, content loss, and frequency loss. Patch loss computes the mean difference between the output and target images at the patch-level instead of pixel-level, thus further addressing the misalignment problem. The content loss assesses their consistency at the feature level, employing a VGG-19^97^ network. Finally, the pyramid loss^62^ is used to preserve consistency at different levels. The authors utilised three datasets to train and evaluate the model’s performance: an in-house dataset of canine lymphoma, BCI^62^, and MIST^44^. They achieved better PSNR, FID, and LPIPS in all three datasets when compared to ASP, Pix2PixHD, pyramid Pix2Pix, cycleGAN and CUT. Evaluation of the CD3 and PAX5 stained in-house dataset by two veterinary Pathologists showed decent similarity between the generated and real images, with clinical accuracies above 60% (average of five diagnostic indicators: staining intensity, cellular localisation, cellular distribution, quantification and morphological correlation).

Li et al.^98^ introduced Confusion-GAN, a cycleGAN-based model that incorporates a patch-level pathology information extractor (PPIE) for H&E image classification, along with a multi-branch discriminator (MBD). An IHC classifier is trained on positive (>1% stained patch area) patch-level labels of the generated IHC, and the loss, combined with the PPIE, results in a term that captures the pathological information of the input patch. The PPIE consists of an encoder that maximises the distance in feature space between the top-k H&E tiles (selected via attention from an ABMIL^99^) of a positive WSI and negative tiles transformed by a BBDM^100^ into positive examples. Based on these embeddings, a classifier is trained to distinguish positive classes from negative ones, which is then combined with the previously mentioned IHC classifier loss. Each generator is associated with two MBDs, which use a discriminator to distinguish real from generated images, while a secondary classifier identifies the generated image by mixing embeddings at a random channel position. In addition to training on an in-house hepatocellular carcinoma dataset, the authors retrained and evaluated their model on the BCI and MIST datasets. Their experiments demonstrate improved FID, LPIPS, and SSIM values compared to Pix2Pix variations and ASP on the MIST dataset. Since these datasets provide tiles rather than WSIs, the authors applied IHC labels using the previously mentioned staining cut-off (or the BCI labels) and transferred them to the corresponding H&E images for use with the PPIE. It is worth mentioning that the authors made a qualitative assessment of the images generated, but only on the internal dataset (for hepatocellular carcinoma).

### Diffusion models

In 2024, He et al.^101^ introduced the pathological stain transfer diffusion (PST-DIFF) model that learns a bidirectional transfer between the source and target domains and then interconnects the latent spaces of both modalities, based on the dual diffusion implicit bridges (DDIBs) method^102^. The authors introduce a pathological consistency constraint through a latent transfer (LT) module to minimise the distance between the latent representations of paired H&E and IHC images. Additionally, they use an asymmetric attention mechanism (AAM) that uses multi-head attention for the source-to-latent conversion and global attention for the latent-to-target conversion. A structural consistency constraint is implemented via a conditional frequency guidance (CFG) module^103^, which conditions the de-noising network on low-frequency information in the early stages and on high-frequency information during the later stages of the reverse process. Training and evaluating their model on the BCI dataset demonstrates better luminance and contrast similarity (LC), source-structural similarity (SS), visual information fidelity (VIF) and histogram correlation metrics compared to GAN models, though performance is lower on PSNR and SSIM. When evaluated on the ACROBAT-HER2 dataset, the model shows decent generalisability. Stain quantification into four categories via ImageJ showed the highest accuracy (72%) for PST-DIFF compared to other models.

With a similar line of work, Li et al.^104^ proposed the histopathological image multi-domain multi-omics translation with diffusion model (His-MMDM). This model uses a diffusion process conditioned on the source (H&E stain) and target (several IHC stains) to generate images, with a U-net performing the backward noise prediction. In addition to IHC stains, the model also allows conditioning on mutations, tissue types, and other class-conditional inputs. The virtual staining model is trained using H&E (27,530 tiles) and 13 IHC stains from an in-house dataset of brain tumour samples, including PgR (2862 tiles) and Ki67 (4846 tiles). While the authors do not conduct a comparison for virtual IHC staining, they report comparable FID-related metrics when evaluating cryosection-to-FFPE translation against a specialised model from Ozyoruk et al.^105^.

## Benchmarking of representative models

**Fig. 7:**
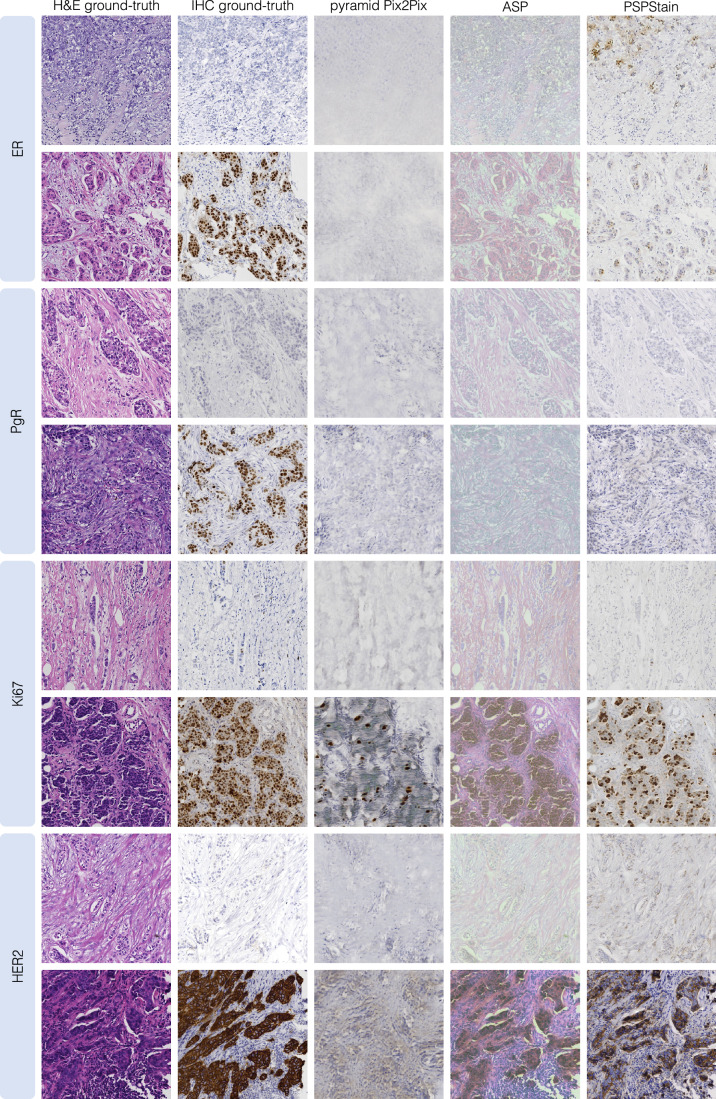
Benchmarking results examples from the MIST dataset, for ER, PgR, Ki67 and HER2 stainings. Images with higher resolution are provided in Supplementary Figs. 1–4.

**Fig. 8:**
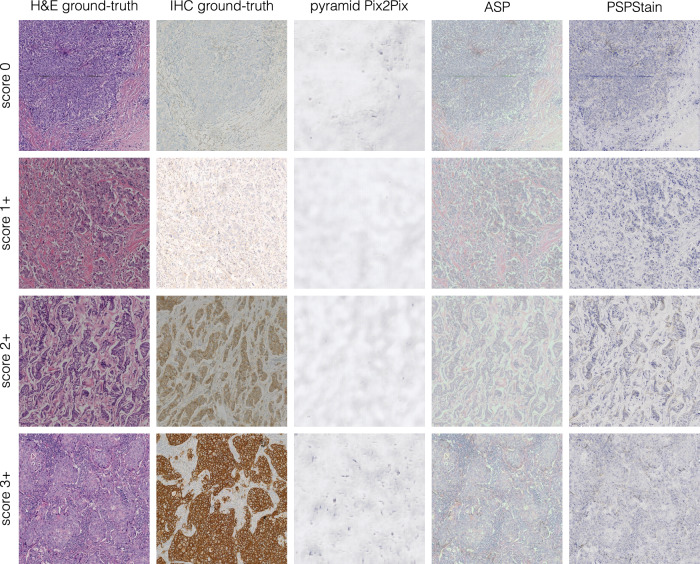
Benchmarking generalisation results on HER2 examples from the BCI dataset. Images with higher resolution are provided in the supplementary Fig. 5.

While a comprehensive comparison of methodologies is usually beyond the scope of a literature review, we present a benchmarking study of selected representative models based on SSIM, PSNR, FID, LPIPS and the Jensen–Shannon distance (JSD) between the distributions of the DAB channel intensities in real and generated IHC images. Accounting for the four breast cancer IHC biomarkers, we focused on models trained and optimised on the MIST dataset and for which code is publicly available. This comparison includes the pyramid Pix2Pix^62^ and ASP^44^ models, which are considered seminal in the virtual staining field, as well as PSPStain^93^, which exemplifies a model that enforces pathological consistency. For a fair comparison, all models were trained on a Nvidia A100 40GB GPU, with all random processes initialised using a common seed. Hyperparameter selection was based on the values reported in the respective articles when disclosed or the default values provided in the code. The train and test sets of MIST were used for model training and validation, respectively, since no validation partition or information about the corresponding slide of each image was provided. The best model was chosen based on the average SSIM value (Table 3 and Fig. 7). Taking advantage of the BCI test set, we assessed the generalisability of the model trained for the HER2 staining (Table 4 and Fig. 8).

**Table 3:** Performance metrics on the MIST test set (used for validation)

IHC marker	Model	LPIPS *↓*	FID *↓*	SSIM *↑*	PSNR *↑*	JSD *↓*
ER	Pyramid Pix2Pix	0.600 ± 0.040	150	**0.380** **±** **0.123**	15.5 ± 3.6	0.900 ± 0.173
	ASP	0.589 ± 0.033	226	0.340 ± 0.108	**15.8** **±** **3.1**	0.884 ± 0.150
	PSPStain	**0.551** **±** **0.032**	**60**	0.317 ± 0.113	14.7 ± 3.5	**0.748** **±** **0.270**
PgR	Pyramid Pix2Pix	0.599 ± 0.035	143	**0.379** **±** **0.127**	15.5 ± 4.0	0.878 ± 0.221
	ASP	0.586 ± 0.031	201	0.366 ± 0.107	**15.6** **±** **3.7**	0.901 ± 0.144
	PSPStain	**0.549** **±** **0.030**	**67**	0.304 ± 0.115	15.1 ± 3.5	**0.822** **±** **0.218**
Ki-67	Pyramid Pix2Pix	0.598 ± 0.035	145	0.377 ± 0.115	15.3 ± 2.7	**0.764** **±** **0.277**
	ASP	0.583 ± 0.033	227	**0.384** **±** **0.114**	**16.5** **±** **2.2**	0.965 ± 0.069
	PSPStain	**0.548** **±** **0.029**	**47**	0.340 ± 0.098	15.6 ± 2.3	0.788 ± 0.249
HER2	Pyramid Pix2Pix	0.609 ± 0.027	154	**0.349** **±** **0.107**	15.3 ± 3.0	0.774 ± 0.221
	ASP	0.580 ± 0.032	177	0.304 ± 0.098	**15.8** **±** **2.8**	0.827 ± 0.187
	PSPStain	**0.556** **±** **0.027**	**54**	0.263 ± 0.096	14.7 ± 2.7	**0.746** **±** **0.254**

For metrics with *↑*, higher values are better, while for metrics with *↓*, lower values are better. The standard deviation reflects the variability of the metric values across all evaluated images. The best metrics for each IHC marker are highlighted in bold.

**Table 4:** Performance metrics for generalisation on the BCI test set (HER2 staining)

Model	LPIPS *↓*	FID *↓*	SSIM *↑*	PSNR *↑*	JSD *↓*
Pyramid Pix2Pix	0.558 ± 0.058	316	**0.624** **±** **0.155**	15.0 ± 5.1	0.748 ± 0.234
ASP	0.543 ± 0.034	205	0.510 ± 0.144	**16.2** **±** **3.3**	**0.732** **±** **0.237**
PSPStain	**0.540** **±** **0.046**	**177**	0.462 ± 0.163	15.6 ± 3.1	0.971 ± 0.068

For metrics with *↑*, higher values are better, while for metrics with *↓*, lower values are better. The standard deviation reflects the variability of the metric values across all evaluated images. The best metrics are highlighted in bold.

From the results on the MIST dataset (Table 3), pyramid Pix2Pix generally yields the highest SSIM values, while ASP achieves the best PSNR values, with minimal differences between the two models. On the other hand, PSPStain outperforms in terms of LPIPS, FID and JSD on the DAB channel intensity histograms. A similar trend can be noticed when evaluating the HER2 models’ generalisability on the BCI test set (Table 4). However, PSPStain shows a significant increase in JSD on the DAB channel, indicating greater dissimilarity between the distributions DAB channel intensities of real and generated images.

A visual inspection of the models’ outputs (Figs. 7 and 8) shows that pyramid Pix2Pix produces highly blurred images with minimal preservation of the morphology in the input H&E images. Since the H&E and IHC images correspond to consecutive tissue sections, their structure and morphology are not perfectly aligned, which explains why the generation of blurred images might bias evaluation metrics like SSIM and PSNR, incorrectly favouring pyramid Pix2Pix as the best-performing model. In contrast, LPIPS, which measures the distance between activations of a network trained to detect human perceptual differences in images, is more robust to discrepancies between the H&E image and its consecutive paired IHC image. Additionally, while ASP consistently outperforms pyramid Pix2Pix in LPIPS values, it is not a definitive indicator of reliable results. In fact, despite the outputs retaining the H&E morphology, they do not exhibit the defining features of an IHC image.

## Discussion

Virtual staining from H&E to IHC of breast biomarkers is a relatively new research direction with unique approaches and challenges. Starting with the first paper in 2021 by Liu et al.^70^, the field has rapidly expanded, with 64% of existing models published just in 2024. Most models are based on the GAN framework and use either a classic cycleGAN architecture or a variation of the Pix2Pix model. The two works introducing both the BCI^62^ and the MIST^44^ datasets have to be highlighted, as they are the *de facto* standard for evaluation and benchmarking. Furthermore, these authors introduced the pyramid Pix2Pix model and its further development, the ASP model, which was popularised using a contrastive objective. Both models serve as inspiration and basic building blocks for several subsequent models, which proves their importance. One of the unique challenges of virtual staining is to keep the pathological information of the source image intact. Thus, recent models have tried to incorporate loss functions that try to account for this by utilising the DAB channel or auxiliary classifiers. So far, no clear winning strategy ensuring that pathological information is correctly preserved has emerged, and thus, we expect further development on that front. With PST-DIFF^101^ and His-MMDM^104^, two diffusion models were published until the end of 2024, and we expect that the use of this framework for virtual staining in breast biomarkers will increase.

While the widespread use of the BCI or MIST datasets for evaluation aids in the comparability of models, it also highlights one pressing problem: the lack of diverse, publicly available, and high-quality datasets. These datasets are a great public resource, but they consist of pairs from consecutive slides, which makes it difficult to assess whether pathological information was actually preserved due to imperfect registration and the fact that cells on one slide might not be present on the consecutive slide. While models such as ASP try to account for this with innovative loss functions, there is an urgent need for public datasets that perform both H&E and IHC staining on the same slide. This re-staining procedure is difficult but has been employed previously for HER2, ER, PR and Ki67^58,106^. Another factor highlighting the need for more high-quality public datasets is the necessity for model generalisability between scanners, study sites, tissue types, and diverse demographics. Generalisability has been neglected so far, and usually, when using external datasets, authors retrain their models. As seen in our benchmarking, this can lead to insufficient results as differences between two datasets can be large. To bring virtual staining models from their infancy to actual use in the clinics, future work should focus more on generalisability and robustness. This should include training models on multiple heterogeneous datasets to account for both H&E and IHC staining variance due to the aforementioned factors. Other approaches, such as training the same model on different stains^107^, may also achieve similar outcomes by learning more robust features. However, when looking at virtual staining for BC biomarkers, there is a lack of guidelines on best practices, such as using stain augmentations, stain normalisation methods or multiple heterogenous datasets^4,61^, which are common for other tasks in computational pathology. Future research should investigate the applicability of these best practices as well as novel ways to ensure the generalisability and robustness of these methods. More in-depth discussions about other hurdles for clinical practice can be found in the excellent general reviews of virtual staining by Bai et al.^8^ and Latonen et al.^6^.

Another challenge in making models accessible and usable is the lack of focus on interpretability and explainability, which, so far, none of the revised works have explored. When building virtual staining models, it is assumed to be possible to predict biomarker status on a cellular level, based on morphology and general patterns in the image. However, Pathologists cannot assess whether a cell is expressing HER2 just based on its appearance in an H&E image. Thus, for (stain-to-stain translation) to work, it must be assumed that there are hidden patterns that a deep learning-based model is picking up. This fundamental assumption is not necessarily true, and more research needs to be conducted to see how models decide which cells to stain and whether it is even possible to perform virtual staining from H&E to IHC, for all biomarkers.

Due to the generative nature of the AI models usually used, model hallucinations should also be taken into account during development^6,108^. In virtual staining, such an effect can be manifested by false positive/negative stained cells or even the hallucination of tissue regions that are not present in the source image but which might aid, for example, in fooling the discriminator in the case of GAN training.

Moreover, determining which model is the best is also not straightforward. Metrics such as SSIM and PSNR, while easy to compute and compare, are flawed for working with imperfectly aligned images and often do not correspond to actual perceptual quality. Metrics such as FID or KID are an improvement for evaluating the distributions of images that a model can generate, but fail at assessing the most important characteristic of a generated image, e.g., whether the pathological information from the source is accurately preserved and highlighted. Some studies approach this issue by calculating metrics on the DAB channel or getting qualitative assessments by Pathologists, but so far, no method has emerged that can claim to be the golden standard for assessing the performance of preserving pathological information in virtual staining.

Our benchmarking shows that more sophisticated models, such as PSPStain, perform worse on classic metrics like SSIM and PSNR, but excel on perceptual-related metrics such as FID and LPIPS. Notably, the inclusion of pathology-related losses seems to help the model generate DAB distributions similar to the target images. However, this does not extend to out-of-domain distributions, and the model fails to generalise. Whether these pathology-related losses provide additional information that could enhance performance on downstream tasks remains uncertain and should be explored in future research. However, the current models still don’t seem to be able to capture the details of each staining and produce visually plausible and realistic results, making any evaluation of performance on downstream tasks unreliable.

Additionally, the benchmarking results highlight the critical need for publicly available datasets comprising H&E and IHC images from the same tissue section. Existing publicly available datasets predominantly consist of image pairs from consecutive slides, which complicates image registration and precludes the possibility of obtaining pixel-perfect alignments. While these datasets facilitate model training, they present significant challenges for model evaluation, as most metrics fail to accurately assess model performance under such conditions.

## Conclusion

This review has focused on generating IHC from H&E, as we believe this to be the most important domain translation task in virtual staining. An emerging trend in computational pathology has been the rise of multi-modal models. While developing these models, for example, integrating spatial transcriptomics with H&E, will most likely lead to better model performance, we believe that they will not be as useful as generating IHC images based on just H&E due to the availability and low cost of generating H&E WSIs in daily clinical practice. Another trend is the rising use of Diffusion Models. In the present review, only two papers utilised Diffusion Models, but we predict that their use will increase due to easier training and higher quality output, as has been observed in other fields and their use in industry. Another hallmark of a maturing field is its progression towards commercialisation, as for example, the main tool of PictorLabs^109^, an American software company dedicated to AI-powered virtual staining technologies. Future studies should focus on the applicability and uptake of virtual staining solutions and address crucial outstanding questions, such as how to best integrate these models into clinical practice.

## Supplementary information

Supplementary Information

## Data Availability

The datasets analysed during the current study are available in the BCI (https://bupt-ai-cz.github.io/BCI/) and MIST (https://github.com/lifangda01/AdaptiveSupervisedPatchNCE) dataset repositories.

## References

[1] Funkhouser, W. K. Pathology: the clinical description of human disease. In *Essential Concepts in Molecular Pathology (Second Edition)*, 177–190 (2020).

[2] Magaki, S., Hojat, S. A., Wei, B., So, A. & Yong, W. H. An introduction to the performance of immunohistochemistry. In *Methods in Molecular Biology*, 289–298 (Springer, 2019). 10.1007/978-1-4939-8935-5_25 PMC6749998 30539453

[3] Veta, M., Pluim, J. P. W., van Diest, P. J. & Viergever, M. A. Breast cancer histopathology image analysis: a review. *IEEE Trans. Biomed. Eng.* **61**, 1400–1411 (2014). 24759275 10.1109/TBME.2014.2303852

[4] Abels, E. et al. Computational pathology definitions, best practices, and recommendations for regulatory guidance: a white paper from the Digital Pathology Association. *J. Pathol.* **249**, 286–294 (2019). 31355445 10.1002/path.5331 PMC6852275

[5] Acs, B., Rantalainen, M. & Hartman, J. Artificial intelligence as the next step towards precision pathology. *J. Intern. Med.* **288**, 62–81 (2020). 32128929 10.1111/joim.13030

[6] Latonen, L., Koivukoski, S., Khan, U. & Ruusuvuori, P. Virtual staining for histology by deep learning. *Trends Biotechnol.* **42**, 1177–1191 (2024). 38480025 10.1016/j.tibtech.2024.02.009

[7] Pillar, N. & Ozcan, A. Virtual tissue staining in pathology using machine learning. *Expert Rev. Mol. Diagn.* **22**, 987–989 (2022). 36440487 10.1080/14737159.2022.2153040

[8] Bai, B. et al. Deep learning-enabled virtual histological staining of biological samples. *Light Sci. Appl.* **12**, 57 (2023). 36864032 10.1038/s41377-023-01104-7 PMC9981740

[9] Kingma, D. P. & Welling, M. Auto-encoding variational Bayes. Preprint at *arXiv*10.48550/arXiv.1312.6114 (2022).

[10] Goodfellow, I. et al. Generative Adversarial Nets. In *Advances in Neural Information Processing Systems—NIPS*, **27**, 2672–2680 (2014).

[11] Zhu, J.-Y., Park, T., Isola, P. & Efros, A. A. Unpaired image-to-image translation using cycle-consistent adversarial networks. In *IEEE International Conference on Computer Vision—ICCV*, 2242–2251 (2017).

[12] Coles, C. E. et al. The Lancet Breast Cancer Commission: tackling a global health, gender, and equity challenge. *Lancet* **399**, 1101–1103 (2022). 35189077 10.1016/S0140-6736(22)00184-2

[13] World Health Organization. *Breast Cancer*. https://www.who.int/news-room/fact-sheets/detail/breast-cancer (2024).

[14] World Health Organization. Global Breast Cancer Initiative Implementation Framework: assessing, strengthening and scaling-up of services for the early detection and management of breast cancer. https://iris.who.int/bitstream/handle/10665/365784/9789240067134-eng.pdf (2023).

[15] Lukong, K. E. Understanding breast cancer—-the long and winding road. *BBA Clin.* **7**, 64–77 (2017). 28194329 10.1016/j.bbacli.2017.01.001 PMC5300293

[16] The Cancer Genome Atlas Network. Comprehensive molecular portraits of human breast tumours. *Nature* **490**, 61–70 (2012). 23000897 10.1038/nature11412 PMC3465532

[17] Goldhirsch, A. et al. Personalizing the treatment of women with early breast cancer: highlights of the St Gallen International Expert Consensus on the Primary Therapy of Early Breast Cancer 2013. *Ann. Oncol.* **24**, 2206–2223 (2013). 23917950 10.1093/annonc/mdt303 PMC3755334

[18] Russnes, H. G., Lingjærde, O. C., Børresen-Dale, A.-L. & Caldas, C. Breast cancer molecular stratification: from intrinsic subtypes to integrative clusters. *Am. J. Pathol.* **187**, 2152–2162 (2017). 28733194 10.1016/j.ajpath.2017.04.022

[19] Spitale, A., Mazzola, P., Soldini, D., Mazzucchelli, L. & Bordoni, A. Breast cancer classification according to immunohistochemical markers: clinicopathologic features and short-term survival analysis in a population-based study from the South of Switzerland. *Ann. Oncol.* **20**, 628–635 (2009). 19074747 10.1093/annonc/mdn675

[20] Coates, A. S. et al. Tailoring therapies—improving the management of early breast cancer: St Gallen International Expert Consensus on the Primary Therapy of Early Breast Cancer 2015. *Ann. Oncol.* **26**, 1533–1546 (2015). 25939896 10.1093/annonc/mdv221 PMC4511219

[21] Curigliano, G. et al. De-escalating and escalating treatments for early-stage breast cancer: the St. Gallen International Expert Consensus Conference on the Primary Therapy of Early Breast Cancer 2017. *Ann. Oncol.* **28**, 1700–1712 (2017). 28838210 10.1093/annonc/mdx308 PMC6246241

[22] Collins, L. *Tumors of the Mammary Gland*. *AFIP Atlas of Tumor and Non-Tumor Pathology* (American Registry of Pathology, 2024).

[23] Rakha, E. A. et al. Updated UK Recommendations for HER2 assessment in breast cancer. *J. Clin. Pathol.* **68**, 93–99 (2015). 25488926 10.1136/jclinpath-2014-202571 PMC4316916

[24] Allison, K. H. et al. Estrogen and progesterone receptor testing in breast cancer: ASCO/CAP guideline update. *J. Clin. Oncol.* **38**, 1346–1366 (2020). 31928404 10.1200/JCO.19.02309

[25] Rakha, E. A. et al. Assessment of predictive biomarkers in breast cancer: challenges and updates. *Pathobiology* **89**, 263–277 (2022). 35728576 10.1159/000525092

[26] Wolff, A. C. et al. Human epidermal growth factor receptor 2 testing in breast cancer: American Society of Clinical Oncology–College of American Pathologists Guideline Update. *J. Clin. Oncol.* **41**, 3867–3872 (2023). 37284804 10.1200/JCO.22.02864

[27] Vieira, A. F. & Schmitt, F. An update on breast cancer multigene prognostic tests-emergent clinical biomarkers. *Front. Med.* **5**, 248 (2018). 10.3389/fmed.2018.00248 PMC6131478 30234119

[28] Erber, R. et al. Molecular subtyping of invasive breast cancer using a PAM50-based multigene expression test-comparison with molecular-like subtyping by tumor grade/immunohistochemistry and influence on oncologist’s decision on systemic therapy in a real-world setting. *Int. J. Mol. Sci.* **23**, 8716 (2022). 35955851 10.3390/ijms23158716 PMC9368794

[29] Hacking, S. M., Yakirevich, E. & Wang, Y. From immunohistochemistry to new digital ecosystems: a state-of-the-art biomarker review for precision breast cancer medicine. *Cancers* **14**, 3469 (2022). 35884530 10.3390/cancers14143469 PMC9315712

[30] Rakha, E. A., Tse, G. M. & Quinn, C. M. An update on the pathological classification of breast cancer. *Histopathology* **82**, 5–16 (2023). 36482272 10.1111/his.14786 PMC10108289

[31] Karaali, C. et al. The clinical and pathological characteristics that differentiate cases with “Low Estrogen Receptor Expression” from triple-negative breast cancer. *Eur. J. Breast Health* **20**, 19 (2024). 38187108 10.4274/ejbh.galenos.2023.2023-6-3 PMC10765462

[32] Colomer, R. et al. It is not time to stop progesterone receptor testing in breast cancer. *J. Clin. Oncol.* **23**, 3868–3869 (2005). 15923595 10.1200/JCO.2005.05.203

[33] Li, J. et al. Expert consensus on the clinical diagnosis and targeted therapy of HER2 breast cancer. *Transl. Breast Cancer Res.* **3**, 30 (2022). 38751529 10.21037/tbcr-22-48 PMC11093007

[34] Nielsen, T. O. et al. Assessment of Ki67 in breast cancer: updated recommendations from the international Ki67 in breast cancer working group. *J. Natl Cancer Inst.* **113**, 808–819 (2021). 33369635 10.1093/jnci/djaa201 PMC8487652

[35] Ramesh, A. et al. Zero-shot text-to-image generation. In *Proceedings of the 38th International Conference on Machine Learning - ICML 2021*, **139**, 8821-8831 (2021).

[36] Rombach, R., Blattmann, A., Lorenz, D., Esser, P. & Ommer, B. High-resolution image synthesis with latent diffusion models. In *2022 IEEE/CVF Conference on Computer Vision and Pattern Recognition—CVPR*, 10674–10685 (2022).

[37] Mirza, M. & Osindero, S. Conditional generative adversarial nets. Preprint at *arXiv*10.48550/arXiv.1411.1784 (2014).

[38] Park, T., Efros, A. A., Zhang, R. & Zhu, J.-Y. Contrastive learning for unpaired image-to-image translation. In *European Conference on Computer Vision - ECCV 2020*, 319–345 (2020).

[39] Sohl-Dickstein, J., Weiss, E. A., Maheswaranathan, N. & Ganguli, S. Deep unsupervised learning using nonequilibrium thermodynamics. In *Proceedings of the 32nd International Conference on Machine Learning - ICML 2015,* **37**, 2256-2265 (2015).

[40] Isola, P., Zhu, J.-Y., Zhou, T. & Efros, A. A. Image-to-Image Translation with Conditional Adversarial Networks. In *2017 IEEE Conference on Computer Vision and Pattern Recognition—CVPR*, 5967–5976 (2017).

[41] Ronneberger, O., Fischer, P. & Brox, T. U-Net: convolutional networks for biomedical image segmentation. In *Medical Image Computing and Computer-Assisted Intervention—MICCAI 2015*, 234–241 (2015).

[42] He, K., Zhang, X., Ren, S. & Sun, J. Deep Residual Learning for Image Recognition. In *2016 IEEE Conference on Computer Vision and Pattern Recognition – CVPR*, 770–778 (2016).

[43] Deng, J. et al. Imagenet: a large-scale hierarchical image database. In *2009 IEEE Conference on Computer Vision and Pattern Recognition—CVPR*, 248–255 (2009).

[44] Li, F., Hu, Z., Chen, W. & Kak, A. Adaptive supervised PatchNCE loss for learning H&E-to-IHC stain translation with inconsistent groundtruth image pairs. In *Medical Image Computing and Computer Assisted Intervention—MICCAI*, 632–641 (2023).

[45] Tomczak, J. M.*-Deep Generative Modeling* (Springer International Publishing, 2024).

[46] Ho, J., Jain, A., & Abbeel, P. Denoising Diffusion Probabilistic Models. In *Advances in Neural Information Processing Systems - NeurIPS,* **33**, 6840-6851 (2020).

[47] Saharia, C. et al. Image super-resolution via iterative refinement. *IEEE Trans. Pattern Anal. Mach. Intell.* **45**, 4713–4726 (2023). 36094974 10.1109/TPAMI.2022.3204461

[48] Zhang, Y. et al. Inversion-based Style Transfer with Diffusion Models. In *2023 IEEE/CVF Conference on Computer Vision and Pattern Recognition—CVPR*, 10146–10156 (2023).

[49] Dhariwal, P. & Nichol, A. Diffusion models beat GANs on image synthesis. In *Proceedings of the 35th International Conference on Neural Information Processing Systems—NIPS*, 8780–8794 (2021).

[50] Müller-Franzes, G. et al. A multimodal comparison of latent denoising diffusion probabilistic models and generative adversarial networks for medical image synthesis. *Sci. Rep.* **13**, 12098 (2023). 37495660 10.1038/s41598-023-39278-0 PMC10372018

[51] Wang, Z., Bovik, A., Sheikh, H. & Simoncelli, E. Image quality assessment: from error visibility to structural similarity. *IEEE Trans. Image Process.* **13**, 600–612 (2004). 15376593 10.1109/tip.2003.819861

[52] Wang, Z., Simoncelli, E. & Bovik, A. Multiscale structural similarity for image quality assessment. In *The 37th Asilomar Conference on Signals, Systems & Computers—ACSSC*, 1398–1402 (2003).

[53] Zhang, R., Isola, P., Efros, A. A., Shechtman, E. & Wang, O. The unreasonable effectiveness of deep features as a perceptual metric. In *2018 IEEE/CVF Conference on Computer Vision and Pattern Recognition—CVPR*, 586–595 (2018).

[54] Heusel, M., Ramsauer, H., Unterthiner, T., Nessler, B. & Hochreiter, S. Gans trained by a two time-scale update rule converge to a local nash equilibrium. In *Proceedings of the 31st International Conference on Neural Information Processing Systems—NIPS*, 6629-6640 (2017).

[55] Szegedy, C., Vanhoucke, V., Ioffe, S., Shlens, J. & Wojna, Z. Rethinking the inception architecture for computer vision. In *2016 IEEE Conference on Computer Vision and Pattern Recognition—CVPR*, 2818–2826 (2016).

[56] Bińkowski, M., Sutherland, D. J., Arbel, M. & Gretton, A. Demystifying MMD GANs. In *International Conference on Learning Representations—ICLR* (2018).

[57] Ruifrok, A. C. et al. Quantification of histochemical staining by color deconvolution. *Anal. Quant. Cytol. Histol.* **23**, 291–299 (2001). 11531144

[58] Martino, F. et al. A deep learning model to predict ki-67 positivity in oral squamous cell carcinoma. *J. Pathol. Inform.* **15**, 100354 (2024). 38148967 10.1016/j.jpi.2023.100354 PMC10750186

[59] Peng, Q. et al. Advancing H&E-to-IHC virtual staining with task-specific domain knowledge for HER2 scoring. In *Medical Image Computing and Computer Assisted Intervention—MICCAI 2024*, 3–13 (2024).

[60] Zeng, B. et al. Semi-supervised PR Virtual Staining for Breast Histopathological Images. In *Medical Image Computing and Computer Assisted Intervention—MICCAI 2022*, 232–241 (2022).

[61] Tellez, D. et al. Quantifying the effects of data augmentation and stain color normalization in convolutional neural networks for computational pathology. *Med. Image Anal.* **58**, 101544 (2019). 31466046 10.1016/j.media.2019.101544

[62] Liu, S. et al. BCI: breast cancer immunohistochemical image generation through pyramid Pix2pix. In *2022 IEEE/CVF Conference on Computer Vision and Pattern Recognition Workshops—CVPRW*, 1814–1823 (2022).

[63] Klein, S., Staring, M., Murphy, K., Viergever, M. A. & Pluim, J. P. W. elastix: a toolbox for intensity-based medical image registration. *IEEE Trans. Med. Imaging* **29**, 196–205 (2010). 19923044 10.1109/TMI.2009.2035616

[64] Akbarnejad, A., Ray, N., Barnes, P. J. & Bigras, G. Predicting Ki67, ER, PR, and HER2 statuses from H&E-stained breast cancer images. Preprint at *arXiv*10.48550/arXiv.2308.01982 (2023).

[65] Qaiser, T. et al. HER2 challenge contest: a detailed assessment of automated HER2 scoring algorithms in whole slide images of breast cancer tissues. *Histopathology* **72**, 227–238 (2018). 28771788 10.1111/his.13333

[66] Academia and Industry Collaboration for Digital Pathology. *AIDPATH DB*. https://mitel.dimi.uniud.it/aidpath-db/app/login.php (2017).

[67] Pilutti, D. et al. An adaptive positivity thresholding method for automated Ki67 hotspot detection (AKHoD) in breast cancer biopsies. *Comput. Med. Imaging Graph.* **61**, 28–34 (2017). 28499621 10.1016/j.compmedimag.2017.04.005

[68] Weitz, P. et al. A multi-stain breast cancer histological whole-slide-image data set from routine diagnostics. *Sci. Data* **10**, 562 (2023). 37620357 10.1038/s41597-023-02422-6 PMC10449765

[69] Borovec, J. et al. ANHIR: automatic non-rigid histological image registration challenge. *IEEE Trans. Med. Imaging* **39**, 3042–3052 (2020). 32275587 10.1109/TMI.2020.2986331 PMC7584382

[70] Liu, S. et al. Unpaired stain transfer using pathology-consistent constrained generative adversarial networks. *IEEE Trans. Med. Imaging* **40**, 1977–1989 (2021). 33784619 10.1109/TMI.2021.3069874

[71] Wodzinski, M. & Müller, H. DeepHistReg: unsupervised deep learning registration framework for differently stained histology samples. *Comput. Methods Prog. Biomed.* **198**, 105799 (2021). 10.1016/j.cmpb.2020.105799 33137701

[72] Zhou, B., Khosla, A., Lapedriza, A., Oliva, A. & Torralba, A. Learning deep features for discriminative localization. In *2016 IEEE Conference on Computer Vision and Pattern Recognition—CVPR*, 2921–2929 (2016).

[73] Kim, J., Kim, M., Kang, H. & Lee, K. H. U-GAT-IT: Unsupervised generative attentional networks with adaptive layer-instance normalization for image-to-image translation. In *International Conference on Learning Representations—ICLR* (2020).

[74] Huang, X., Liu, M.-Y., Belongie, S. & Kautz, J. Multimodal unsupervised image-to-image translation. In *European Conference on Computer Vision—ECCV 2018*, 179–196 (2018).

[75] Ma, J. & Chen, H. Efficient supervised pretraining of swin-transformer for virtual staining of microscopy images. *IEEE Trans. Med. Imaging* **43**, 1388–1399 (2024). 38010933 10.1109/TMI.2023.3337253

[76] Liu, Z. et al. Swin transformer: hierarchical vision transformer using shifted windows. In *2021 IEEE/CVF International Conference on Computer Vision—ICCV*, 9992–10002 (2021).

[77] He, K. et al. Masked autoencoders are scalable vision learners. In *Proceedings of the IEEE/CVF Conference on Computer Vision and Pattern Recognition—CVPR*, 16000–16009 (2022).

[78] Xie, Z. et al. SimMIM: a simple framework for masked image modeling. In *Proceedings of the IEEE/CVF Conference on Computer Vision and Pattern Recognition*, 9653–9663 (2022).

[79] Christiansen, E. M. et al. In silico labeling: predicting fluorescent labels in unlabeled images. *Cell* **173**, 792–803 (2018). 29656897 10.1016/j.cell.2018.03.040 PMC6309178

[80] Charbonnier, P., Blanc-Feraud, L., Aubert, G. & Barlaud, M. Two deterministic half-quadratic regularization algorithms for computed imaging. In *Proceedings of 1st IEEE International Conference on Image Processing—ICIP*, 168–172 (1994).

[81] Baldeon-Calisto, M. et al. DeepSIT: deeply supervised framework for image translation on breast cancer analysis. In *IEEE 13th International Conference on Pattern Recognition Systems—ICPRS)*, 1–7 (2023).

[82] Wang, T.-C. et al. High-Resolution Image Synthesis and Semantic Manipulation with Conditional GANs. In *IEEE/CVF Conference on Computer Vision and Pattern Recognition—CVPR*, 8798–8807 (2018).

[83] Liu, S. et al. *Breast Cancer Immunohistochemical Image Generation Challenge*. bci.grand-challenge.org (2024).

[84] Ma, Y. et al. Dsff-gan: A novel stain transfer network for generating immunohistochemical image of endometrial cancer. *Comp. Biol. Med.* **170**, 108046 (2024). 10.1016/j.compbiomed.2024.108046 38325211

[85] Ding, K., Ma, K., Wang, S. & Simoncelli, E. P. Image quality assessment: unifying structure and texture similarity. *IEEE Trans. Pattern Anal. Mach. Intell.* **44**, 2567–2581 (2022). 33338012 10.1109/TPAMI.2020.3045810

[86] Wei, L., Hua, S., Zhang, S. & Zhang, X. DeReStainer: H&E to IHC pathological image translation via decoupled staining channels. In -(eds Mukhopadhyay, A., Oksuz, I., Engelhardt, S., Mehrof, D. & Yuan, Y.) *Lecture Notes in Computer Science*, Deep Generative Models. DGM4MICCAI 2024, 1–10 (Springer, 2025).

[87] Lin, T.-Y., Goyal, P., Girshick, R., He, K. & Dollár, P. Focal loss for dense object detection. In *2017 IEEE International Conference on Computer Vision—ICCV*, 2999–3007 (2017).

[88] Guan, X., Wang, Y., Lin, Y., Li, X. & Zhang, Y. Unsupervised multi-domain progressive stain transfer guided by style encoding dictionary. *IEEE Trans. Image Process.* **33**, 767–779 (2024). 38198253 10.1109/TIP.2024.3349866

[89] Zhang, R. et al. MVFStain: multiple virtual functional stain histopathology images generation based on specific domain mapping. *Med. Image Anal.* **80**, 102520 (2022). 35810588 10.1016/j.media.2022.102520

[90] Ustinova, E. & Lempitsky, V. Learning deep embeddings with histogram loss. In *Proceedings of the 30th International Conference on Neural Information Processing Systems—NIPS*, 4177-4185 (2016).

[91] Qu, L. et al. Advancing H&E-to-IHC stain translation in breast cancer: a multi-magnification and attention-based approach. In *2024 IEEE International Conference on Cybernetics and Intelligent Systems and IEEE International Conference on Robotics, Automation and Mechatronics—CIS-RAM*, 441–446 (2024).

[92] Zhang, K., Liang, J., Van Gool, L. & Timofte, R. Designing a practical degradation model for deep blind image super-resolution. In *IEEE International Conference on Computer Vision*, 4791–4800 (2021).

[93] Chen, F. et al. Pathological Semantics-Preserving Learning for H&E-to-IHC Virtual Staining. In *Medical Image Computing and Computer Assisted Intervention—MICCAI 2024*, 384–394 (2024).

[94] Li, Y., Guan, X., Wang, Y. & Zhang, Y. Exploiting supervision information in weakly paired images for IHC virtual staining. In *Medical Image Computing and Computer Assisted Intervention—MICCAI 2024*, 113–122 (2024).

[95] Wang, S., Zhang, Z., Yan, H., Xu, M. & Wang, G. Mix-domain contrastive learning for unpaired H&E-to-IHC stain translation. In *2024 IEEE International Conference on Image Processing—ICIP*, 2982–2988 (2024).

[96] Zhang, W. et al. High-resolution medical image translation via e20614patch alignment-based bidirectional contrastive learning. In *Medical Image Computing and Computer Assisted Intervention—MICCAI 2024*, 178–188 (2024).

[97] Simonyan, K. & Zisserman, A. Very deep convolutional networks for large-scale image recognition. Preprint at *arXiv*10.48550/arXiv.1409.1556 (2015).

[98] Li, J. et al. Virtual immunohistochemistry staining for histological images assisted by weakly-supervised learning. In *2024 IEEE/CVF Conference on Computer Vision and Pattern Recognition—CVPR*, 11259–11268 (2024).

[99] Ilse, M., Tomczak, J. & Welling, M. Attention-based deep multiple instance learning. In *35th International Conference on Machine Learning—ICML 2018*, 3376–3391 (2018).

[100] Li, B., Xue, K., Liu, B. & Lai, Y.-K. BBDM: Image-to-image translation with brownian bridge diffusion models. In *2023 IEEE/CVF Conference on Computer Vision and Pattern Recognition—CVPR*, 1952–1961 (2023).

[101] He, Y. et al. PST-Diff: achieving high-consistency stain transfer by diffusion models with pathological and structural constraints. *IEEE Trans. Med. Imaging* **43**, 3634–3647 (2024). 39024079 10.1109/TMI.2024.3430825

[102] Su, X., Song, J., Meng, C. & Ermon, S. Dual diffusion implicit bridges for image-to-image translation. Preprint at *arXiv*10.48550/arXiv.2203.08382 (2023).

[103] Choi, J., Kim, S., Jeong, Y., Gwon, Y. & Yoon, S. ILVR: conditioning method for denoising diffusion probabilistic models. In *2021 IEEE/CVF International Conference on Computer Vision—ICCV*, 14347–14356 (2021).

[104] Li, Z. et al. His-MMDM: multi-domain and multi-omics translation of histopathology images with diffusion models. *medRxiv*10.1101/2024.07.11.24310294 (2024).

[105] Ozyoruk, K. B. et al. A deep-learning model for transforming the style of tissue images from cryosectioned to formalin-fixed and paraffin-embedded. *Nat. Biomed. Eng.* **6**, 1407–1419 (2022). 36564629 10.1038/s41551-022-00952-9

[106] Gamble, P. et al. Determining breast cancer biomarker status and associated morphological features using deep learning. *Comm. Med.* **1**, 14 (2021). 10.1038/s43856-021-00013-3 PMC9037318 35602213

[107] Pati, P. et al. Accelerating histopathology workflows with generative AI-based virtually multiplexed tumour profiling. *Nat. Mach. Intell.* **6**, 1077–1093 (2024). 39309216 10.1038/s42256-024-00889-5 PMC11415301

[108] Waqas, A. et al. Revolutionizing digital pathology with the power of generative artificial intelligence and foundation models. *Lab. Investig.* **103**, 100255 (2023). 37757969 10.1016/j.labinv.2023.100255

[109] Levin, M. et al. Multi-modal spatial analysis of classic Hodgkin lymphoma microenvironments utilizing multiplex immunofluorescence and virtual staining. *J. Immunother. Cancer* **12**, 1195 (2024).

[110] Duan, G., Cao, Y., Guo, W., Cui, L. & Liu, Z. A virtual staining method for immunohistochemical images of breast cancer. In *2023 16th International Congress on Image and Signal Processing, BioMedical Engineering and Informatics—CISP-BMEI)*, 1–5 (2023).

[111] Huang, S. et al. Tc-cyclegan: improved cyclegan with texture constraints for virtual staining of pathological images. In *Proceedings of the 3rd International Conference on Bioinformatics and Intelligent Computing—BIC’23*, 147–152 (2023).

[112] Liu, L. et al. MGGAN: a multi-generator generative adversarial network for breast cancer immunohistochemical image generation. *Heliyon* **9**, e20614 (2023). 37860562 10.1016/j.heliyon.2023.e20614 PMC10582479

[113] Wu, S. & Xu, S. HcGAN: harmonic conditional generative adversarial network for efficiently generating high-quality IHC images from H&E. *Heliyon* **10**, e37902 (2024). 39678164 10.1016/j.heliyon.2024.e37902 PMC11639370

[114] Hu, J. et al. ULViT-GAN: Advancing stain transfer in H&E and IHC pathology images with a UNet-like vision-transformer GAN. In *Proceedings of the 3rd International Conference on Computer, Artificial Intelligence and Control Engineering—CAICE’24*, 524-528 (2024).

[115] Jia, Y., Duan, G., Song, Y., Ye, L. & Liu, Z. DTNet: Dual-encoder generative adversarial network for generating breast cancer immunohistochemical images. In *2024 5th International Conference on Computer Vision, Image and Deep Learning—CVIDL*, 937–942 (2024).

[116] Ji, C. et al. Transformation from hematoxylin-and-eosin staining to Ki-67 immunohistochemistry digital staining images using deep learning: experimental validation on the labeling index. *J. Med. Imaging* **11**, 047501 (2024). 10.1117/1.JMI.11.4.047501 PMC11287056 39087085

