# Supplementary material for: H&E to IHC virtual staining methods in breast cancer: an overview and benchmarking
Source: NPJ Digit Med. 2025 Jul 2;8:384. doi: 10.1038/s41746-025-01741-9 (PMC12222792; doi:10.1038/s41746-025-01741-9)
Supplement: Supplementary file 1 — Supplementary Information [file 41746_2025_1741_MOESM1_ESM.pdf]

Supplementary Figure 1

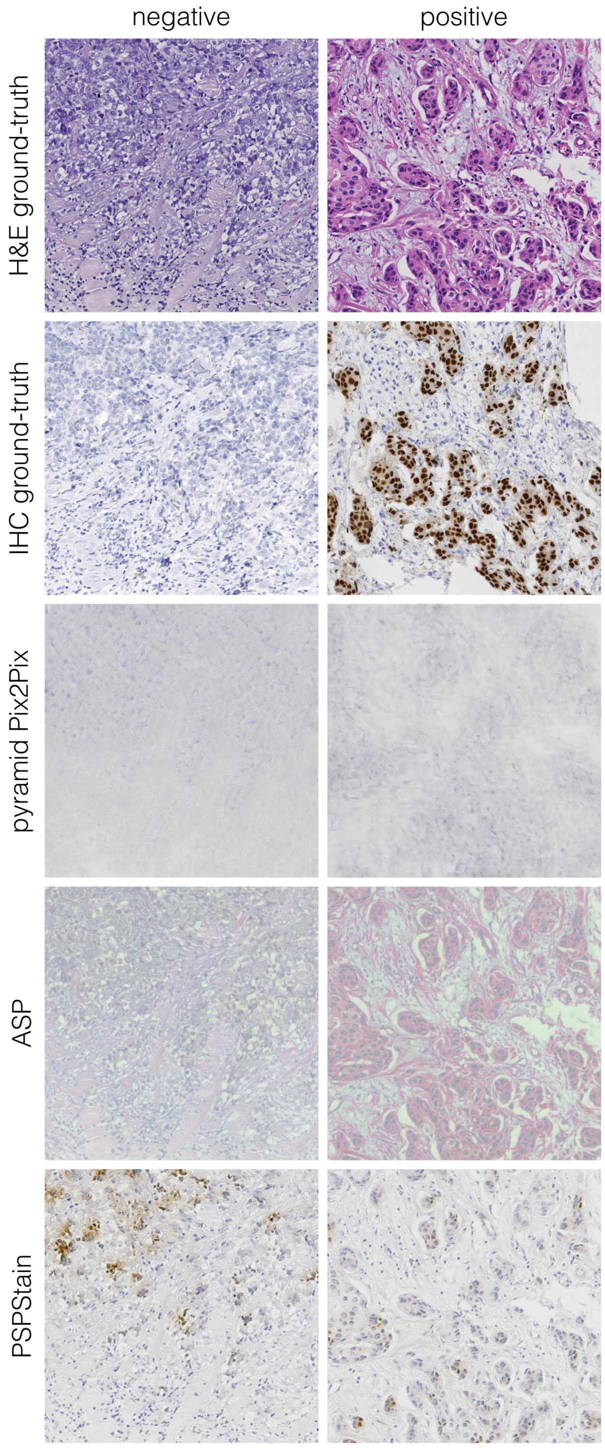

Benchmarking results examples from the MIST dataset for the ER staining.

Supplementary Figure 2

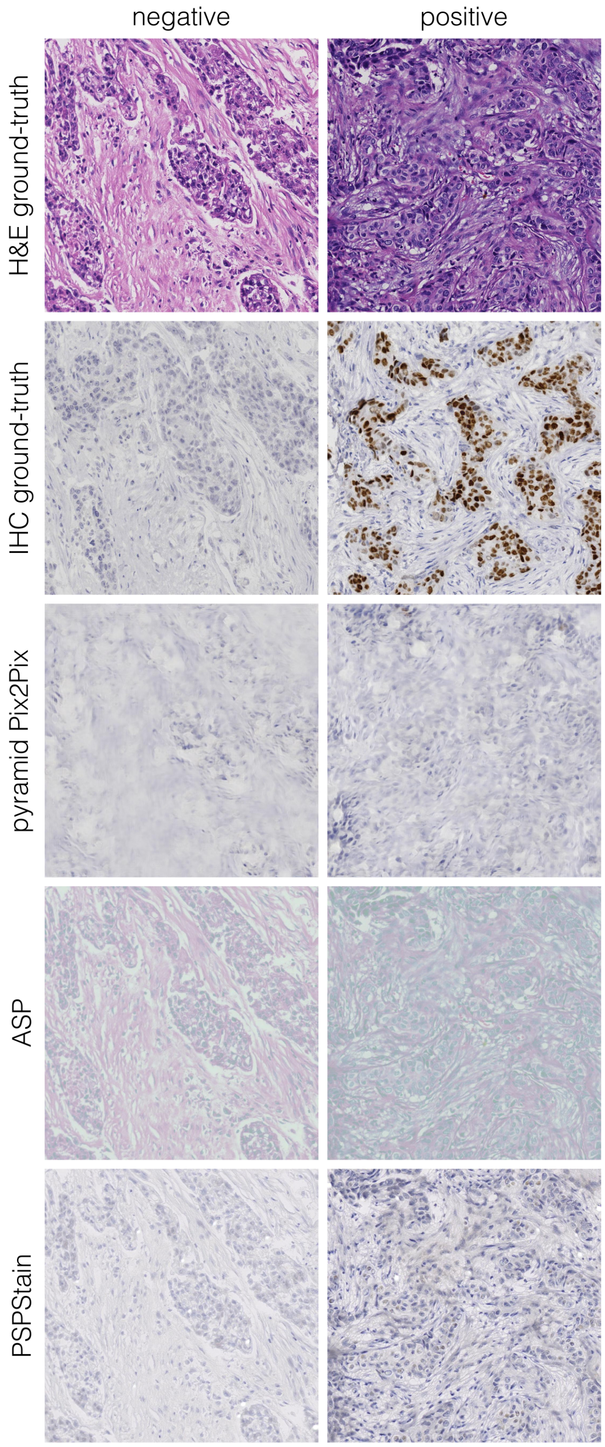

Benchmarking results examples from the MIST dataset for the PgR staining.

Supplementary Figure 3

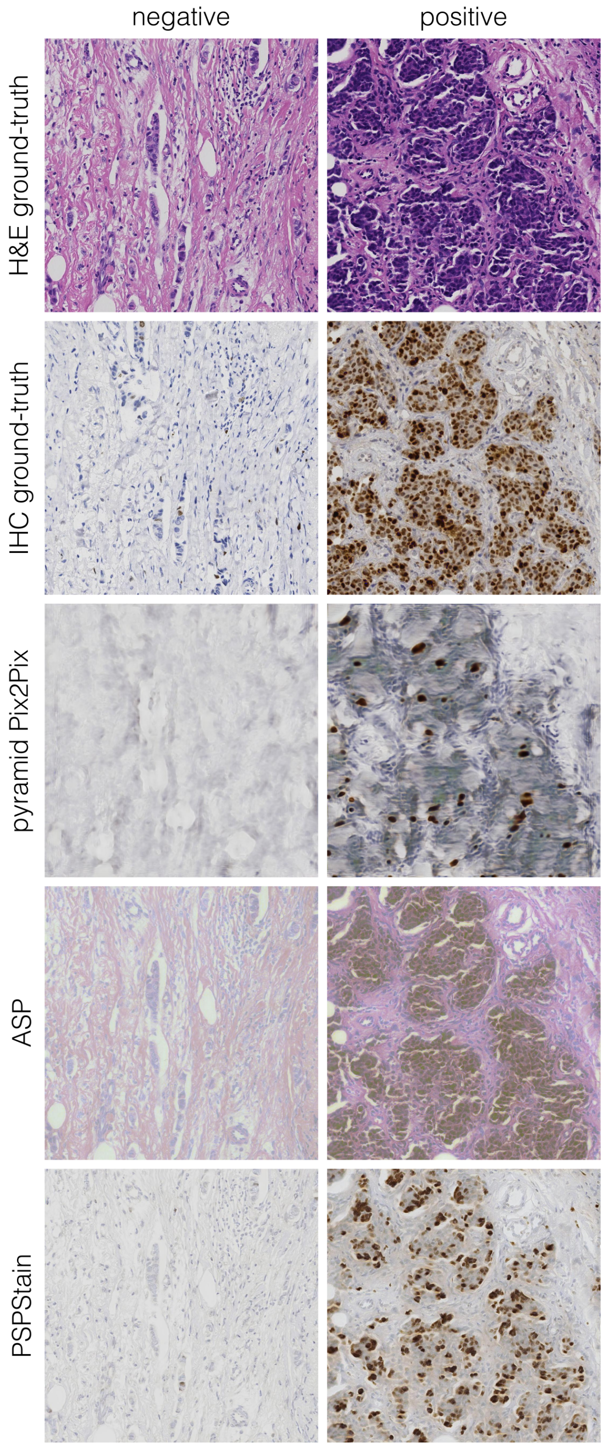

Benchmarking results examples from the MIST dataset for the Ki67 staining.

Supplementary Figure 4

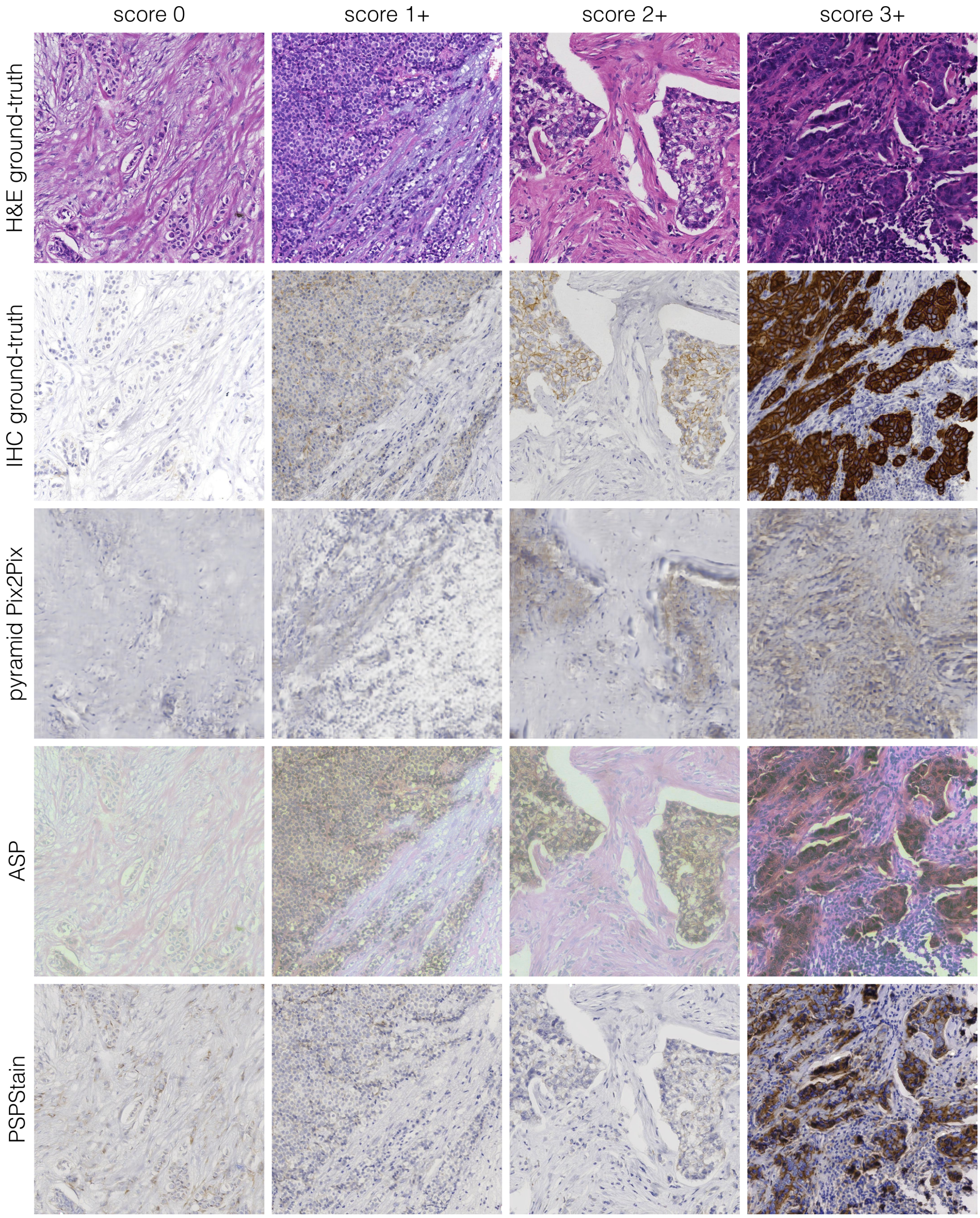

Benchmarking results examples from the MIST dataset for the HER2 staining.

Supplementary Figure 5

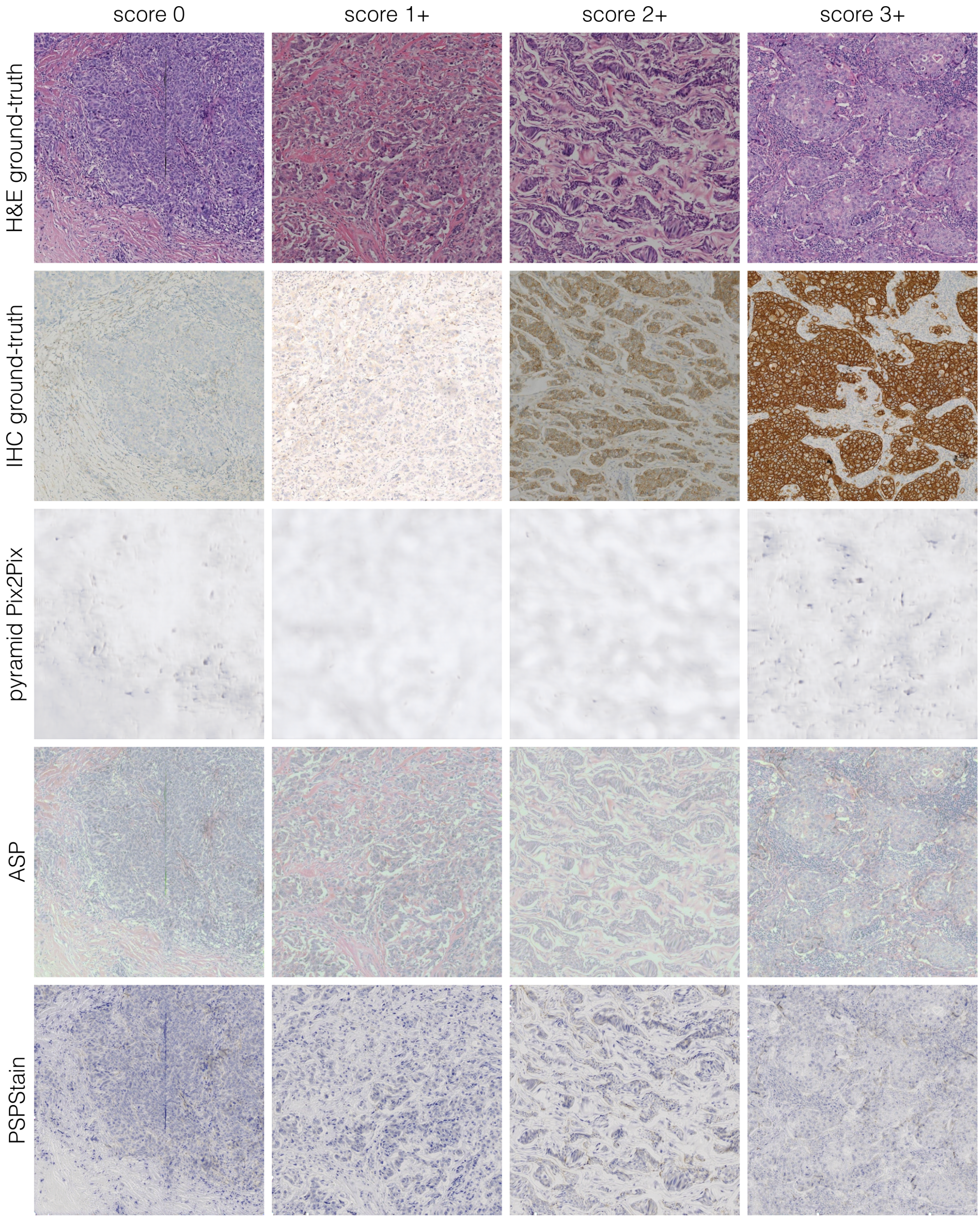

Benchmarking generalisation results on HER2 examples from the BCI dataset.
